# BTK inhibitor–induced defects in human neutrophil effector activity against *Aspergillus fumigatus* are restored by TNF-**α**

**DOI:** 10.1172/jci.insight.176162

**Published:** 2024-05-07

**Authors:** Diego A. Vargas-Blanco, Olivia W. Hepworth, Kyle J. Basham, Patricia Simaku, Arianne J. Crossen, Kyle D. Timmer, Alex Hopke, Hannah Brown Harding, Steven R. Vandal, Kirstine N. Jensen, Daniel J. Floyd, Jennifer L. Reedy, Christopher Reardon, Michael K. Mansour, Rebecca A. Ward, Daniel Irimia, Jeremy S. Abramson, Jatin M. Vyas

**Affiliations:** 1 Division of Infectious Diseases, Department of Medicine, Massachusetts General Hospital, Boston, Massachusetts, USA.; 2 Harvard Medical School, Boston, Massachusetts, USA.; 3 BioMEMS Resource Center, Massachusetts General Hospital, Boston, Massachusetts, USA.; 4 Shriners Hospital for Children, Boston, Massachusetts, USA.; 5 Beth Israel Deaconess Medical Center, Boston, Massachusetts, USA.; 6 Center for Lymphoma, Mass General Cancer Center, Boston, Massachusetts, USA.

**Keywords:** Immunology, Infectious disease, Cancer, Fungal infections, Neutrophils

## Abstract

Inhibition of Bruton’s tyrosine kinase (BTK) through covalent modifications of its active site (e.g.*,* ibrutinib [IBT]) is a preferred treatment for multiple B cell malignancies. However, IBT-treated patients are more susceptible to invasive fungal infections, although the mechanism is poorly understood. Neutrophils are the primary line of defense against these infections; therefore, we examined the effect of IBT on primary human neutrophil effector activity against *Aspergillus fumigatus*. IBT significantly impaired the ability of neutrophils to kill *A*. *fumigatus* and potently inhibited reactive oxygen species (ROS) production, chemotaxis, and phagocytosis. Importantly, exogenous TNF-α fully compensated for defects imposed by IBT and newer-generation BTK inhibitors and restored the ability of neutrophils to contain *A*. *fumigatus* hyphal growth. Blocking TNF-α did not affect ROS production in healthy neutrophils but prevented exogenous TNF-α from rescuing the phenotype of IBT-treated neutrophils. The restorative capacity of TNF-α was independent of transcription. Moreover, the addition of TNF-α immediately rescued ROS production in IBT-treated neutrophils, indicating that TNF-α worked through a BTK-independent signaling pathway. Finally, TNF-α restored effector activity of primary neutrophils from patients on IBT therapy. Altogether, our data indicate that TNF-α rescued the antifungal immunity block imposed by inhibition of BTK in primary human neutrophils.

## Abstract 2

BTK inhibitors impair neutrophil effector functions in response to *Aspergillus fumigatus,* and exogenous TNFα rescues BTK inhibitor-induced antifungal immunity blockade.

## Graphical abstract

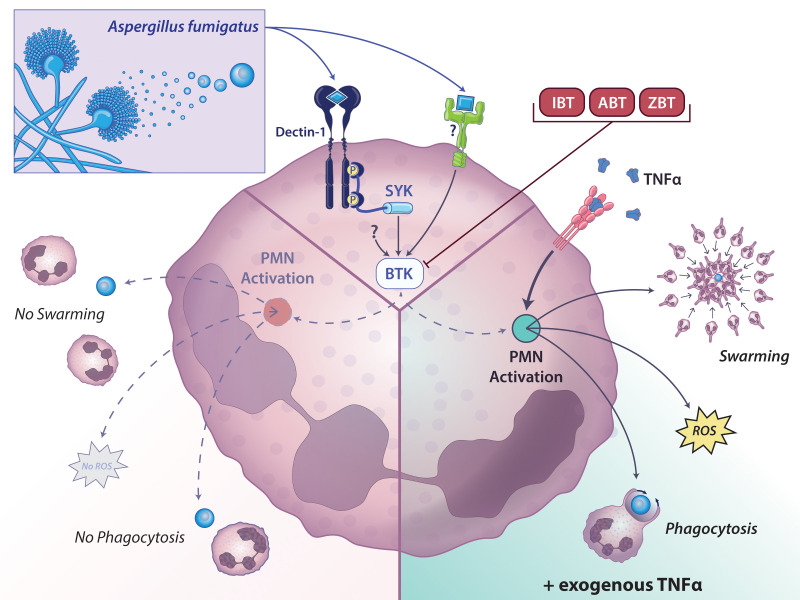

## Introduction

Invasive fungal infections are dreaded complications for those with compromised immune systems, including patients with cancer (e.g., leukemia, lymphoma) and solid-organ and hematopoietic stem cell transplant recipients. The fungal pathogen *Aspergillus spp*. causes a spectrum of diseases, including asthma, chronic infection, and invasive disease. Invasive fungal infections carry elevated mortality rates in these high-risk patients, despite the availability of antifungals (1–4), demonstrating the critical role of the innate immune system as the first line of defense against these devastating infections (5, 6).

As the first responders in fungal infections, neutrophils exert antifungal activity through multiple effector functions, including swarming, phagocytosis, and reactive oxygen species (ROS) production. Activation of neutrophil pattern recognition receptors triggers these effector functions and subsequent cytokine secretion. However, a reduced ability to produce neutrophils or neutrophil dysfunction occurs in many immunosuppressed individuals, contributing to an elevated risk of invasive fungal infections, including invasive aspergillosis. Tyrosine kinases are critical to neutrophil effector function in antifungal immunity (7–9). *Aspergillus* cell wall carbohydrates trigger intracellular signaling cascades and effector functions through spleen tyrosine kinase (Syk) (10, 11). Bruton’s tyrosine kinase (BTK), a kinase downstream of Syk, mediates antifungal response in innate immune cells, including neutrophils (12). While these kinases are critical in antifungal immunity, small-molecule inhibitors targeting these molecules are effective therapeutics for B cell malignancies and chronic graft-versus-host disease (13–16).

Unfortunately, BTK inhibitor therapy amplifies the risk of invasive infections, including fungal pathogens, particularly in dissemination to the central nervous system (CNS) (15, 17–20). Although BTK inhibitors (e.g., acalabrutinib [ABT], ibrutinib [IBT], zanubrutinib [ZBT]) improve outcomes in multiple subtypes of B cell lymphoma and leukemia, BTK, and other Tec protein tyrosine kinases signal diverse cellular processes in immune cell lineages (e.g., macrophages, neutrophils, γδ T cells) (21–24). These BTK inhibitors impair the function of immune cells critical to host defense against invading pathogens through the suppression of proinflammatory cytokines, dampened killing capacity, and blunted ROS production (19, 25–32). Indeed, the irreversible inhibitor of BTK, IBT, quickly reduces BTK phosphorylation at the Tyr^551^ and Tyr^223^ sites and has been linked to defects in murine neutrophils when responding to *Aspergillus* *fumigatus* (29, 31). The effect of BTK inhibition on neutrophil effector functions remains incompletely understood (33).

Here, we demonstrate the deleterious effect of 3 BTK inhibitors (IBT, ABT, and ZBT) on the antifungal effector functions of human neutrophils, including chemotaxis, phagocytosis, and ROS production. Given that genes related to the TNF signaling pathways were the most differentially expressed in IBT-treated neutrophils, we tested the hypothesis that TNF-α could bypass the block imposed by BTK inhibition. We show that exogenous TNF-α improves BTK inhibitor–associated defects, restoring the neutrophil ability to control *A*. *fumigatus* in healthy neutrophils treated with BTK inhibitors as well as in neutrophils from IBT-treated patients. We demonstrate that the restorative effect of exogenous TNF-α occurs via transcription-independent signaling. Taken together, these data indicate that exogenous TNF-α acts as a signaling molecule in neutrophils, rapidly compensating for BTK inhibitor–imposed defects in response to *A*. *fumigatus*.

## Results

### IBT inhibited neutrophil effector activity against A. fumigatus.

**Figure 1:**
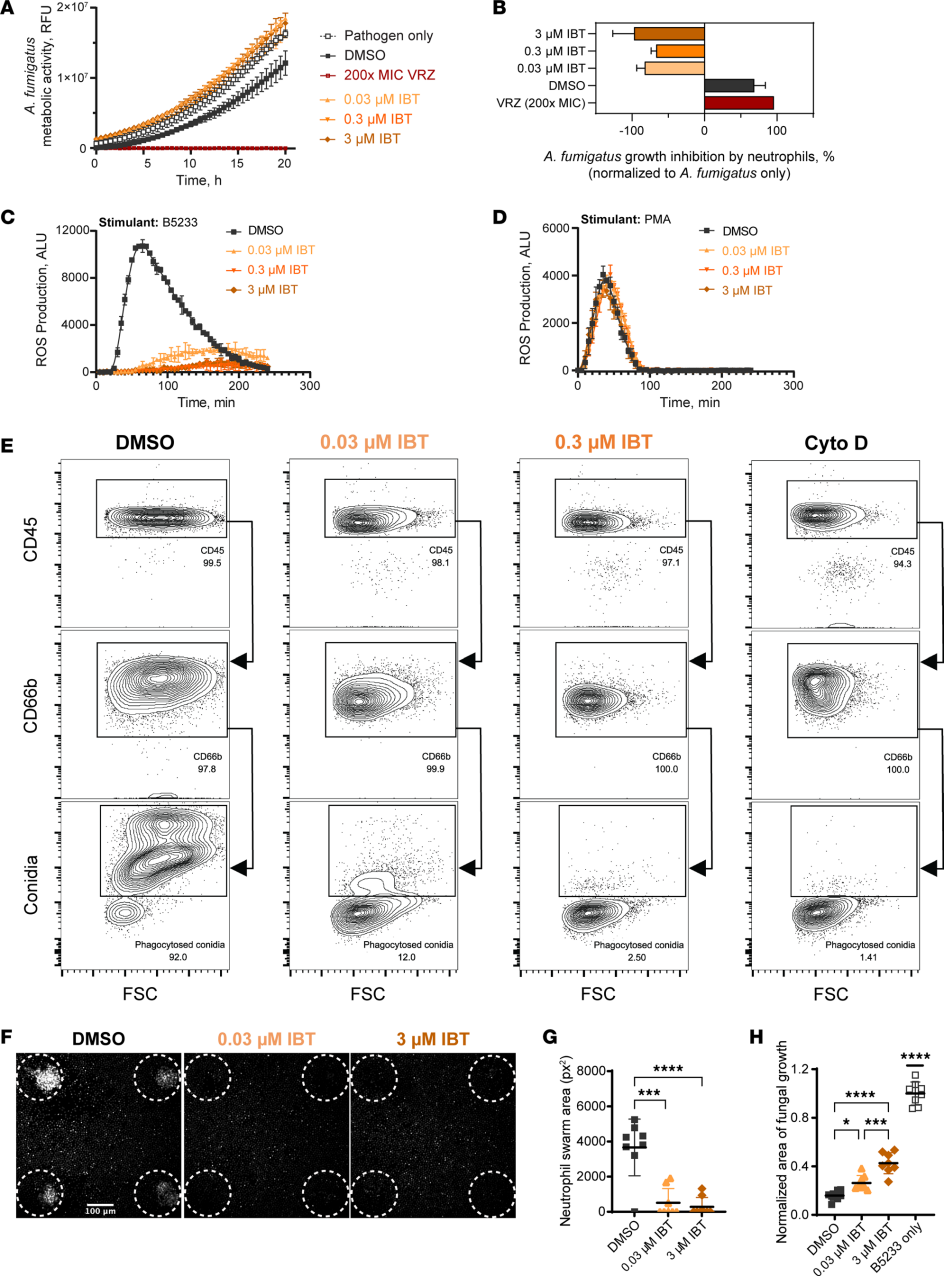
IBT inhibition dampened human neutrophil effector activity against *A*. *fumigatus*. (**A**) Metabolic activity of *A*. *fumigatus* B5233 strain measured using resazurin. Human neutrophils were pretreated for 4 hours (4h) with IBT and stimulated with *A*. *fumigatus* (MOI:0.25) for 5h. Data are shown as mean ± SD, *n* = 3; data are representative of at least 3 independent experiments. (**B**) Percentages of growth inhibition derived from **A** using linear regression analysis in a Gompertz fit. Data are shown as 95% CI, *n* = 3. Ordinary 1-way ANOVA and Tukey’s multiple-comparison test with a single pooled variance demonstrated a *P* < 0.0001 for all IBT treatments versus DMSO alone. (**C** and **D**) Human neutrophils were treated for 4h with IBT or DMSO and then stimulated with 1 mg/mL *A*. *fumigatus* B5233 strain heat-killed hyphal elements (**C**) or 1 μg/mL PMA (**D**). ROS production was measured by chemiluminescence using lucigenin. Data are shown as mean ± SD, *n* = 3. (**E**) Human neutrophils were treated with IBT or DMSO for 4h and incubated with Af488-labeled *A*. *fumigatus* B5233 strain (conidia^+^) swollen spores (MOI: 10). A subset of neutrophils was pretreated with 20 μM of cytochalasin D (Cyto D). The displayed percentage of phagocytic neutrophils (CD45-AF700^+^CD66b-APC^+^conidia-AF488^+^) was estimated based on the total number of viable neutrophils (CD45-AF700^+^CD66b-APC^+^). A minimum of 10,000 viable CD66b-APC^+^ events were recorded. (**F**–**H**) Human neutrophils were treated with IBT or DMSO for 4h before coincubation with *A*. *fumigatus* B5233 strain. Representative microscopy panels from the swarming assay showing neutrophil swarm formations 200 minutes (min) after coincubation, white circles depict areas seeded with *A*. *fumigatus* (**F**). Area of human neutrophil swarm 200 min after coincubation with *A*. *fumigatus* seeded spores (**G**). Area of fungal growth per cluster on swarming array slides after 16h, normalized to *A*. *fumigatus* growth without neutrophils (**H**). Data are shown as mean ± SD, *n* = 8. Ordinary 1-way ANOVA and Tukey’s multiple-comparison test with a single pooled variance. *P < 0.05; ***P < 0.001; ****P < 0.0001. For all panels, data are representative of at least 3 independent experiments.

To evaluate the hypothesis that BTK inhibition of neutrophils affect antifungal immune response against *A*. *fumigatus*, we sought to determine the effect of BTK inhibition on neutrophil effector functions including killing, ROS production, phagocytosis, and swarming by neutrophils when challenged with *A*. *fumigatus*. Primary human neutrophils treated ex vivo with IBT at a physiologically relevant concentration (19, 34) (0.3 μM) or 10-fold higher or lower concentrations failed to kill *A*. *fumigatus* in contrast to neutrophils treated with solvent control (0.1% DMSO), as shown by a resazurin-based metabolic assay (Figure 1A). These data were confirmed by calculating the rate of growth inhibition of *A*. *fumigatus* when compared with the *A*. *fumigatus* growth alone (Figure 1B). These results demonstrate that IBT-treated neutrophils failed to control *A*. *fumigatus* growth as compared with solvent-treated neutrophils.

We next examined the effects on ROS production in primary neutrophils using the same doses as above. Consistent with the metabolic activity assay, IBT-treated neutrophils produced less ROS in response to heat-killed *A*. *fumigatus* hyphae when compared with DMSO-treated neutrophils (Figure 1C). These BTK inhibitor–induced effects on ROS production were not strain specific, and IBT blocked β-glucan–coated bead–induced (the agonist for Dectin-1 signaling) ROS production (Supplemental Figure 1, A–D; supplemental material available online with this article; https://doi.org/10.1172/jci.insight.176162DS1). As a control, we examined the effect of BTK inhibition on Dectin-1 expression in primary human neutrophils as loss of expression of Dectin-1 could be a trivial explanation for these findings. Dectin-1 expression was not altered in IBT-treated neutrophils (Supplemental Figure 1E). To examine whether these effects blocked all induced ROS production, we stimulated IBT-treated neutrophils with PMA, a NADPH oxidase inducer. PMA in the presence of IBT generated ROS similar to the solvent control (Figure 1D), suggesting that IBT-associated ROS defects were specific to ligands found on *A*. *fumigatus*. We examined intracellular ROS production to determine if this process was also sensitive to BTK inhibition. IBT potently reduced the amount of intracellular ROS as determined by flow cytometry (Supplemental Figure 2). These data indicate that IBT blocked both extracellular and intracellular ROS production.

Since pathogen-associated molecular pattern molecules can trigger an increase of neutrophilic phagocytic activity (35), we sought to determine whether BTK inhibitor effect included phagocytosis. We measured neutrophil phagocytosis of *A*. *fumigatus* by flow cytometry using Alexa Fluor 488–labeled (AF488-labeled) conidia. Neutrophils were gated as the double positive CD45^+^CD66b^+^ subpopulation, and evidence of phagocytosis was defined as CD45^+^CD66b^+^Af488^+^. Neutrophil phagocytosis of *A*. *fumigatus* conidia was severely impaired by IBT in a dose-dependent manner when compared with solvent-treated neutrophils (Figure 1E). To rule out stochastic associations of conidia and neutrophils at a superficial level, we used cytochalasin D, an actin polymerization inhibitor, in parallel treatments for each condition tested. In the presence of cytochalasin D, CD45^+^CD66b^+^AF488^+^ events were below 1.35% for solvent-treated neutrophils (Figure 1E), with similar values for all other neutrophil treatments (Supplemental Figure 3). These results indicate that IBT-treated human neutrophils were impaired in their phagocytic capacity as compared with solvent-treated neutrophils.

We next leveraged a neutrophil swarming assay (36) to determine how coordinated chemotaxis to the site of infection and containment of fungal growth may be affected by BTK inhibition. We observed significantly impaired neutrophil swarming over 200 minutes toward *A*. *fumigatus* in IBT-treated neutrophils compared with the solvent control (Figure 1, F and G). In addition, we demonstrated that IBT-treated neutrophils were less able to contain fungal growth compared with solvent-treated neutrophils 16 hours after coincubation of *A*. *fumigatus* (Figure 1H).

### TNF-α compensated IBT-induced defects in neutrophils against A. fumigatus.

**Figure 2:**
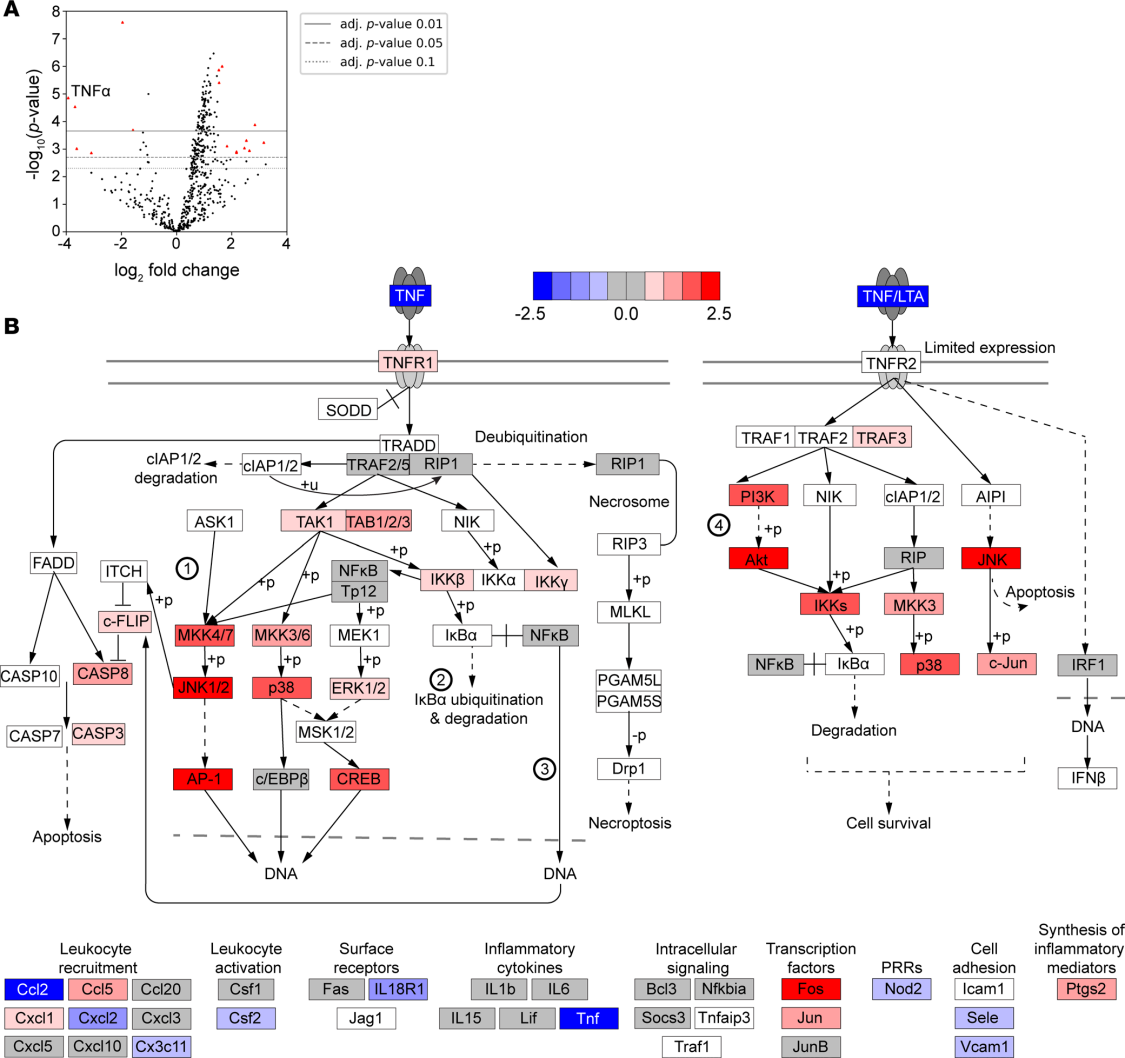
IBT induced downstream upregulation of the TNF-α pathway in human neutrophils. (**A**) Volcano plot for DEGs in neutrophils treated with 0.3 μM IBT versus DMSO (4.5h, unstimulated). DEGs based on log_2_ fold change and *P*_adj_ < 0.05. FDRs were calculated using the Benjamini-Yekutieli method with 3 biological replicates per condition. Red and blue dots represent upregulated and downregulated genes, respectively. (**B**) TNF-α KEGG pathway was created for all probed genes for IBT-treated neutrophils versus DMSO. Genes in white boxes are genes not included in the nCounter panel. Numbers in circles represent pathways: (1) MAPK signaling pathway; (2) ubiquitin-mediated proteolysis; (3) NF-κB signaling pathway; and (4) PI3K/Akt signaling pathway.

To better understand how BTK affected the neutrophil immune response against *A*. *fumigatus*, we assessed signaling pathways affected by IBT treatments at the transcript level. We collected RNA from unstimulated neutrophils treated with either 0.3 μM IBT or solvent control for 4 hours and assessed the expression of 773 host response genes. Using NanoString nCounter, we detected 18 differentially expressed genes (DEGs) in IBT- versus solvent-treated unstimulated neutrophils (Figure 2A, and Table 1). Interestingly, *TNF* was the top hit and was downregulated by a log_2_ fold-change of 4, closely followed by *CD274*, whose product PD-L1 has been positively correlated with TNF-α production (37, 38). Moreover, *RAC2*, important for neutrophil granule exocytosis (39) and TNF-α–mediated ROS production (40), was found to be upregulated. Given the role of multiple DEGs in TNF-α signaling pathways, we next examined upregulated and downregulated genes in the TNF-α pathway using a KEGG map (Figure 2B). The analysis revealed that the genes *ADGRG3*, *ALPL*, *CR1*, *ERN1*, *FOS*, *IL1RAP*, *IL1RL1*, *MAP2K4*, *PIK3CB*, *RAC2*, *TIMP2*, and *TME140* were upregulated or relatively unchanged. Downregulation of *APOL6*, *CD274*, *FBXO6*, *GBP1*, *STAT1*, and *TNF* occurred in IBT-treated neutrophils.

**Figure 3:**
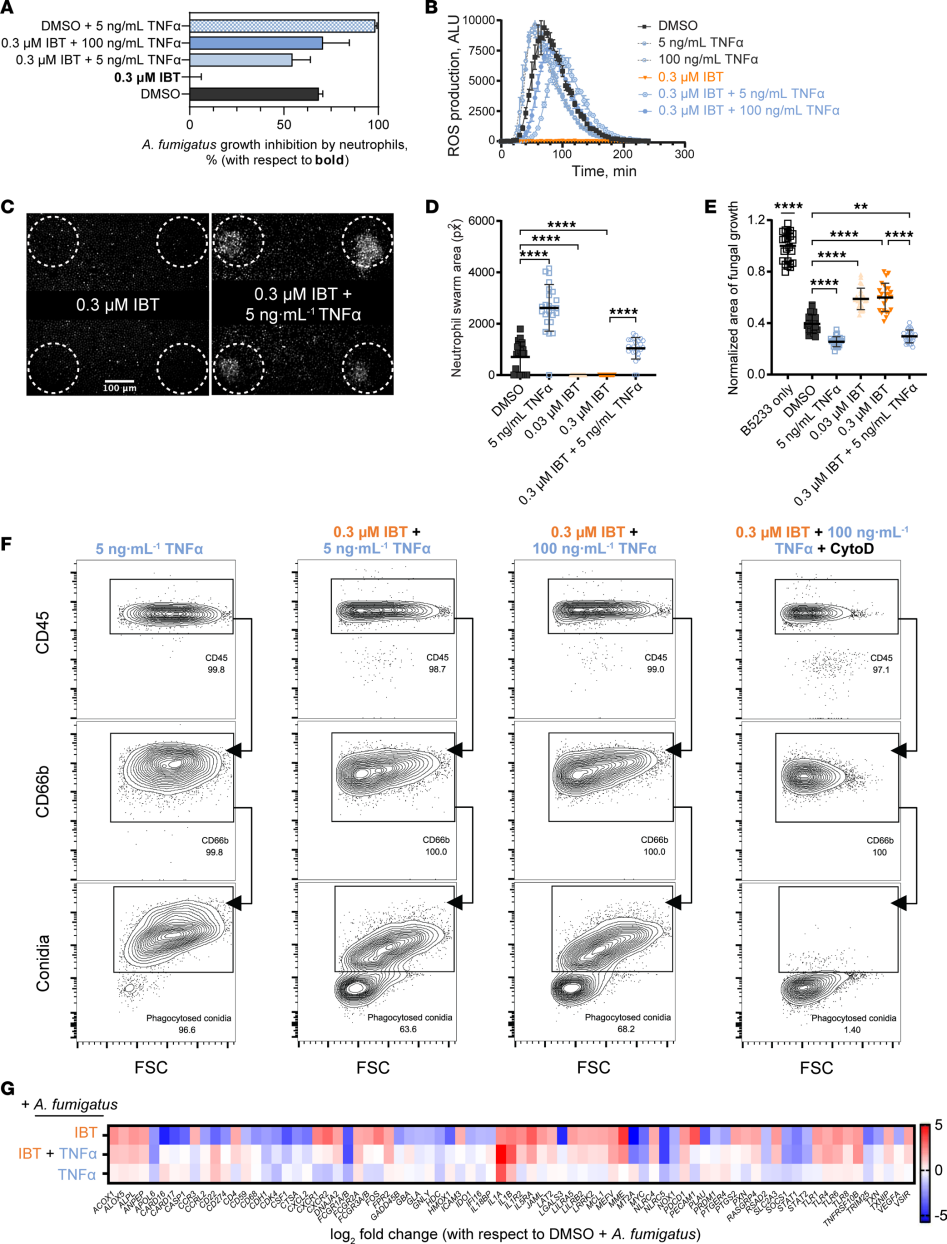
TNF-α rescued IBT-induced immune defects in neutrophils against *A*. *fumigatus*. Human neutrophils were treated with 0.03 μM IBT, 0.3 μM IBT, or DMSO for 30 min followed by a 4h incubation with TNF-α and coincubated with *A*. *fumigatus* B5233 strain for all figure panels. For all panels, data are representative of at least 3 independent experiments. (**A**) Neutrophils were incubated with *A*. *fumigatus* (MOI:0.25) for 5h, and metabolic activity was measured by resazurin assay. Data calculated through time course study (see raw data in the Supporting Data Values file) and panel represent the output from linear regression analysis using Gompertz fit with percentages of growth inhibition of *A*. *fumigatus* by neutrophils in reference to IBT-treated neutrophils. Data are shown as 95% CI, *n* = 3. Ordinary 1-way ANOVA and Tukey’s multiple-comparison test with a single pooled variance demonstrated a *P* < 0.001 for all TNF-α treatments versus IBT alone. (**B**) Neutrophils were stimulated with 1 mg/mL *A*. *fumigatus* heat-killed hyphae. ROS production was measured by chemiluminescence using lucigenin. Data are shown as mean ± SD, *n* = 3. (**C**) Microscopy panels showing neutrophils swarm formations 200 min after coincubation. (**D**) Area of neutrophil swarm after 200 min. (**E**) Area of fungal growth normalized to the growth of *A*. *fumigatus* without neutrophils after 16h. Data are shown as mean ± SD, *n* = 24. Ordinary 1-way ANOVA and Tukey’s multiple-comparison test with a single pooled variance. **P < 0.01; ****P < 0.0001. (**F**) Neutrophils were coincubated with AF488-labeled *A*. *fumigatus* swollen spores (MOI: 10). The displayed percentage of phagocytic neutrophils (CD45-AF700^+^CD66b-APC^+^conidia-AF488^+^) was estimated based on the total number of viable neutrophils (CD45-AF700^+^CD66b-APC^+^). At minimum, 10,000 viable CD66b-APC^+^ events were recorded. (**G**) Heatmap for DEG based on log_2_ fold change (1.5 < log_2_ fold change < –1.5) and a *P*_adj_ < 0.05. FDR was calculated using the Benjamini-Yekutieli method with 3 biological replicates per condition. RNA from neutrophils coincubated for 5h with *A*. *fumigatus* B5233 strain (MOI: 2.5).

Analysis of transcriptional changes in IBT-treated neutrophils revealed that the TNF signaling pathway was the most affected. We hypothesized that exogenous TNF-α could rescue the immune defects in these neutrophils. Most TNF-α in inflammatory conditions are from heterologous sources (e.g., macrophages, dendritic cells), with a small fraction made from neutrophils. To address their contribution, we quantified soluble TNF-α by ELISA using the supernatant of *A*. *fumigatus*–stimulated neutrophils. Indeed, TNF-α levels were 45% lower in IBT-treated cells compared with the solvent control (Supplemental Figure 4A). To test the hypothesis that exogenous TNF-α can restore neutrophil activity against *A*. *fumigatus*, we stimulated IBT-treated neutrophils with recombinant TNF-α, and we then challenged them with *A*. *fumigatus*. At both 5 ng/mL and 100 ng/mL, TNF-α restored effector activity against *A*. *fumigatus* to levels comparable with those of competent neutrophils, as demonstrated by growth inhibition (Figure 3A) and ROS production (Figure 3B and Supplemental Figures 4 and 5). Similarly, TNF-α promoted neutrophil swarming in IBT-treated neutrophils, recapitulating those of control neutrophil treatments (Figure 3, C–E). TNF-α also restored the phagocytic activity of 0.3 μM IBT-treated neutrophils (2.36% phagocytic activity; Figure 1E) compared with 63.6% and 68.2% when 5 ng/mL or 100 ng/mL TNF-α were added, respectively (Figure 3F and Supplemental Figure 5C). We then examined the transcription signature of IBT-treated neutrophils stimulated with *A*. *fumigatus* with and without exogenous TNF-α. Out of 773 genes examined by NanoString nCounter, 79 were DEG in IBT-treated versus solvent control-treated neutrophils stimulated with *A*. *fumigatus*, 65 of which were compensated (genes not significantly dysregulated for IBT + TNF-α versus solvent control) by 5 ng/mL TNF-α (Figure 3G). Taken together, our data indicate that TNF-α, at doses as low as 5 ng/mL, compensated for IBT-induced defects in neutrophils.

In addition to TNF-α, we tested the effect of IFN-γ, G-CSF, IL-1β, and IL-8, on neutrophils treated with 0.3 μM and 3 μM IBT. The effects of GM-CSF on neutrophil function following BTK inhibition is discussed in Desai et al. (41). However, IFN-γ, G-CSF, IL-1β, and IL-8 did not restore neutrophil effector activity but rather further exacerbated the IBT-associated defects for killing capacity against *A*. *fumigatus* (Supplemental Figure 6A). Importantly, growing *A*. *fumigatus* in the presence of IBT or any of these cytokines alone did not alter the pathogen’s basal metabolic activity (data not shown). While the killing capacity was not compensated by these cytokines, G-CSF mildly improved extracellular ROS production. Similarly, IFN-γ, IL-1β, and IL-8 showed a modest increase (Supplemental Figure 6B). Neither TNF-α nor all other tested cytokines elicited neutrophil ROS production in the absence of a stimulant. Additionally, neutrophil swarming and phagocytosis defects were not improved by exogenous IFN-γ, G-CSF, IL-1β, or IL-8 in IBT-treated neutrophils (Supplemental Figure 6, C–E). These data indicate that TNF-α, specifically, restored the defects caused by BTK inhibition on human neutrophil effector activity.

### TNF-α improved effector function defects imposed by other BTK inhibitors.

**Figure 4:**
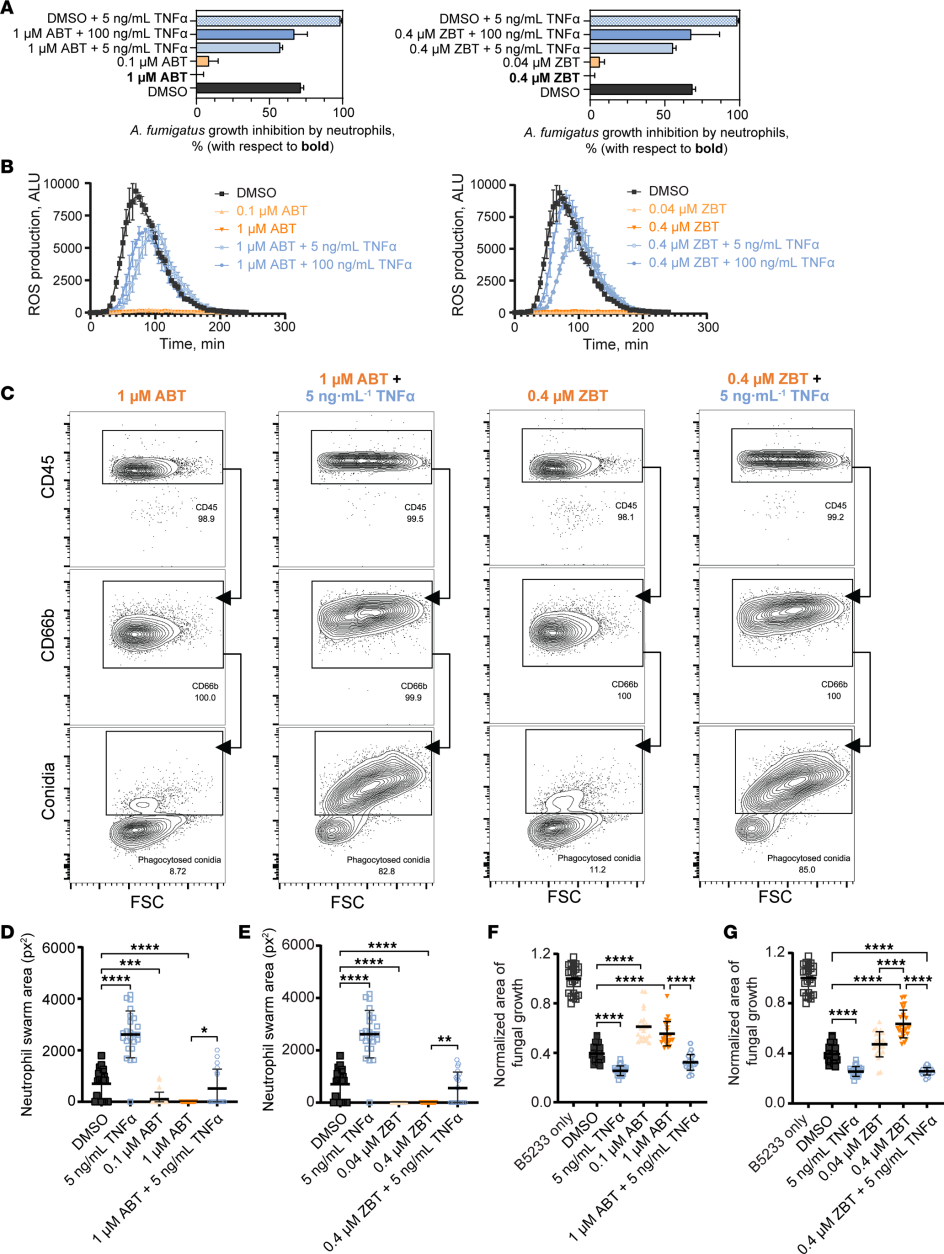
TNF-α restored defects caused by multiple BTK inhibitors on neutrophil immune activity against *A*. *fumigatus*. Human neutrophils were treated with ABT, ZBT, or DMSO for 30 min followed by a 4h incubation with TNF-α and coincubated with *A*. *fumigatus* B5233 strain for all figure panels. For all panels, data are representative of at least 3 independent experiments. (**A**) Neutrophils were incubated with *A*. *fumigatus* (MOI: 0.25) for 5h, and metabolic activity was measured using a resazurin assay. Data calculated through time course study (see raw data in the Supporting Data Values file) and panel represent the output from linear regression analysis using Gompertz fit with percentages of growth inhibition of *A*. *fumigatus* by neutrophils in reference to neutrophils treated with the respective BTK inhibitor. Data are shown as 95% CI, *n* = 3. Ordinary 1-way ANOVA and Tukey’s multiple-comparison test with a single pooled variance demonstrated a *P* < 0.001 for all IBT treatments versus BTK inhibitor (ABT or ZBT) alone. (**B**) Neutrophils were incubated with 1 mg/mL *A*. *fumigatus* heat-killed hyphae. ROS production was measured by chemiluminescence using lucigenin. Data are shown as mean ± SD, *n* = 3. (**C**) Neutrophils treated with ABT (left 2 panels) or ZBT (right 2 panels) were coincubated with labeled *A*. *fumigatus* swollen spores (MOI: 10). The displayed percentage of phagocytic neutrophils (CD45-AF700^+^CD66b-APC^+^conidia-AF488^+^) was estimated based on the total number of viable neutrophils (CD45-AF700^+^CD66b-APC^+^). At minimum, 10,000 viable CD66b-APC^+^ events were recorded. (**D**–**G**) Swarming assay was measured by confocal microscopy view of *A*. *fumigatus* conidia spots after 200 min. Area of neutrophil swarm after 200 min for neutrophils treated with ABT (**D**) or ZBT (**E**). Area of fungal growth per cluster on swarming array slides normalized to the growth of *A*. *fumigatus* without neutrophils after 16h, for neutrophils treated with ABT (**F**) or ZBT (**G**). Treatment controls correspond to the same swarming array experiment (**D**–**G**). Data are shown as mean ± SD, *n* = 24. Ordinary 1-way ANOVA and Tukey’s multiple-comparison test with a single pooled variance. *P < 0.05; **P < 0.01; ***P < 0.001; ****P < 0.0001.

Patients treated with IBT carry an increased risk for invasive fungal infections (15). However, patients on newer agents in this class rarely report significant invasive fungal infections (42–46). It remains unclear whether these agents behave differently with respect to *A*. *fumigatus*–specific neutrophil effect activity. To determine if other FDA-approved BTK inhibitors affected antifungal immunity, we used ABT and ZBT, newer-generation BTK inhibitors with reported decreased off-target activity (47, 48). Using the growth-inhibition measurement, both drugs at physiologically relevant concentrations (1 μM for ABT and 0.4 μM for ZBT) (49–52) or 10-fold below disrupted immunological mechanisms implicated in *Aspergillus* defense (Figure 4A), confirming that BTK inhibition dampened the neutrophil response against *A*. *fumigatus*. Therefore, we considered whether TNF-α could compensate the specific defects imposed by ABT and ZBT in neutrophils. We measured *A*. *fumigatus* killing, ROS production, phagocytosis, and swarming in ABT- and ZBT-treated neutrophils. These experiments revealed similar outcomes to those elicited by IBT, all of which TNF-α rescued to similar levels as the solvent controls (Figure 4, A–G, and Supplemental Figure 7). Taken together, our observations indicate a class-effect of BTK inhibitors that is not limited to a specific drug in this family of chemotherapeutic agents.

### Restorative capacity of exogenous TNF-α was transcription independent.

**Figure 5:**
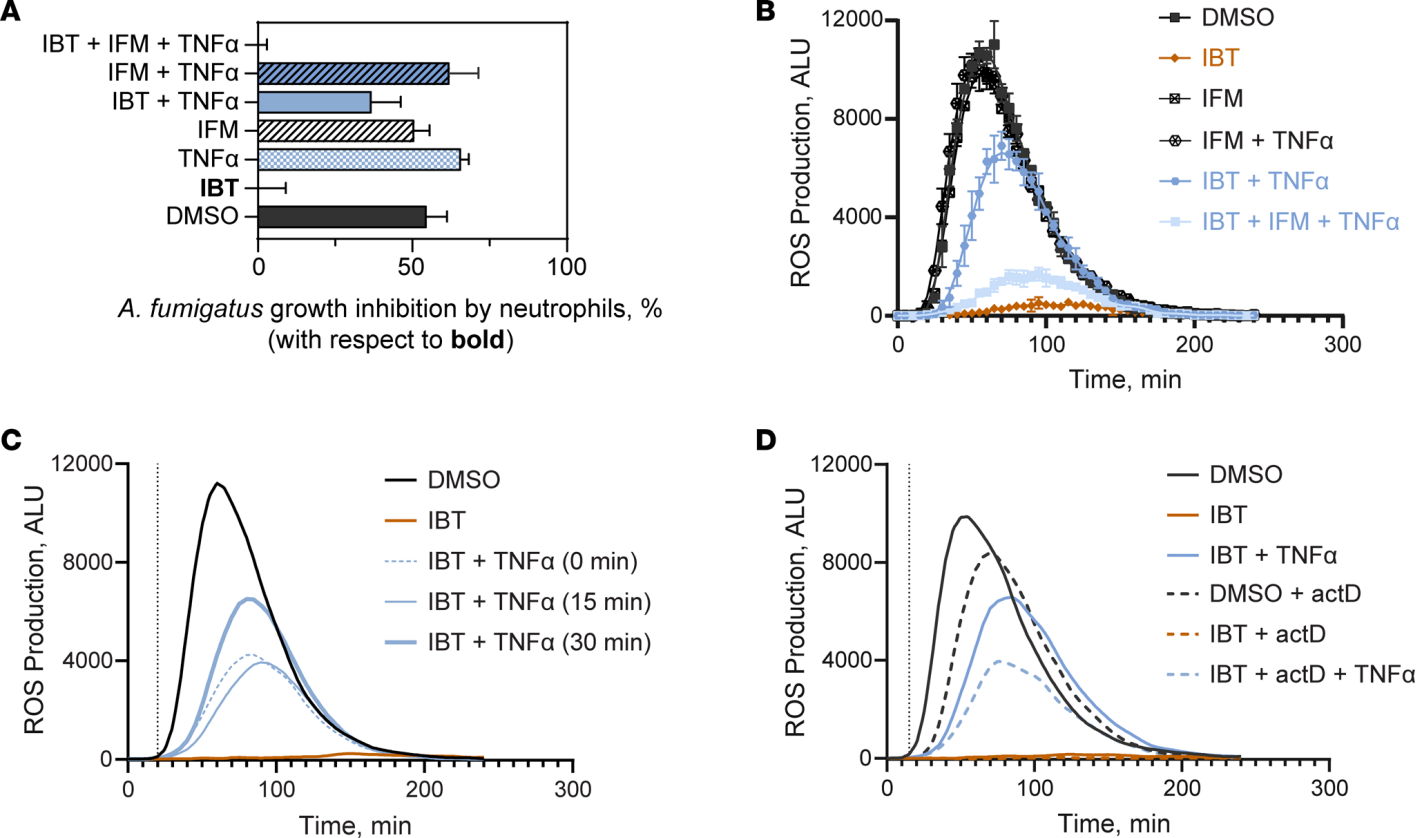
Restorative activity of exogenous TNF-α signals independent of transcription. (**A**) Neutrophils were incubated with *A*. *fumigatus* B5233 strain (MOI: 2.5) for 5h, and metabolic activity was estimated by fluorescence. Data calculated through time course study (see raw data in the Supporting Data Values file) and panel represent the output from linear regression analysis using Gompertz fit with data shown as 95% CI, *n* = 3. Ordinary 1-way ANOVA and Tukey’s multiple-comparison test with a single pooled variance demonstrated a *P* < 0.001 for TNF-α alone, IFM alone, and in combination with IBT treatments versus IBT alone and *P* = 0.0004 for IBT + TNF-α versus IBT alone. (**B**) ROS production in IBT-treated neutrophils incubated with 25 μg/mL IFM in the presence of exogenous TNF-α and coincubated with 1 mg/mL *A*. *fumigatus* heat-killed hyphae. Data are shown as mean ± SD, *n* = 3; data are representative from at least 3 independent experiments. (**C**) Neutrophils were treated with 0.3 μM IBT for 30 min followed by 5 ng/mL TNF-α for the time indicated. To better visualize the starting point of ROS production (black dotted line, 20 min), only the trend but not the time points are shown. (**D**) Neutrophils were treated with DMSO or 0.3 μM IBT for 30 min followed by 1 μg/mL actD for 15 min and by 5 ng/mL TNF-α for 1h. ROS production was measured after stimulation with 1 mg/mL *A*. *fumigatus* heat-killed hyphae. The black dotted line represents the starting point of ROS production (15 min) upon stimulation with *A*. *fumigatus* for treatments containing actD.

Since IBT treatment impaired TNF-α production in neutrophils stimulated with *A*. *fumigatus*, we sought to determine whether endogenously produced TNF-α contributed to the ability of neutrophils to respond to *A*. *fumigatus*. We treated neutrophils with infliximab (IFM), a monoclonal antibody to TNF-α, prior to stimulation with *A*. *fumigatus* and demonstrated no changes in pathogen killing or ROS production compared with the solvent control (Figure 5, A and B). Moreover, adding IFM to IBT-treated neutrophils prior to the addition of TNF-α showed no change in pathogen killing efficiency. However, there was modest ROS production, probably caused by partial activation of the TNF-α receptor.

To assess whether the rescue of neutrophil effector activity by TNF-α required de novo transcriptional activity, we assessed whether exogenous TNF-α utilizes preexisting signaling pathways. We treated neutrophils with IBT or solvent control for 30 minutes, followed by TNF-α immediately before stimulation with *A*. *fumigatus* (0 minutes), 15 minutes, or 30 minutes. TNF-α rescued ROS production even when the cytokine was added immediately before stimulation, with the starting signal detected 20 minutes after stimulation (Figure 5C), demonstrating a swift response prior to expected transcriptional changes. To address directly the role of transcription in this process, neutrophils treated with IBT for 30 minutes were exposed to actinomycin D (actD), a potent transcription inhibitor (53), for 15 minutes and supplemented with TNF-α. Compensation of ROS production by TNF-α occurred even in the absence of transcription (Figure 5D). These data indicate that TNF-α acted through BTK-independent signaling pathways to promote ROS production, without the need for transcription.

### Exogenous TNF-α rescued defects in neutrophils from patients undergoing treatment with IBT.

**Figure 6:**
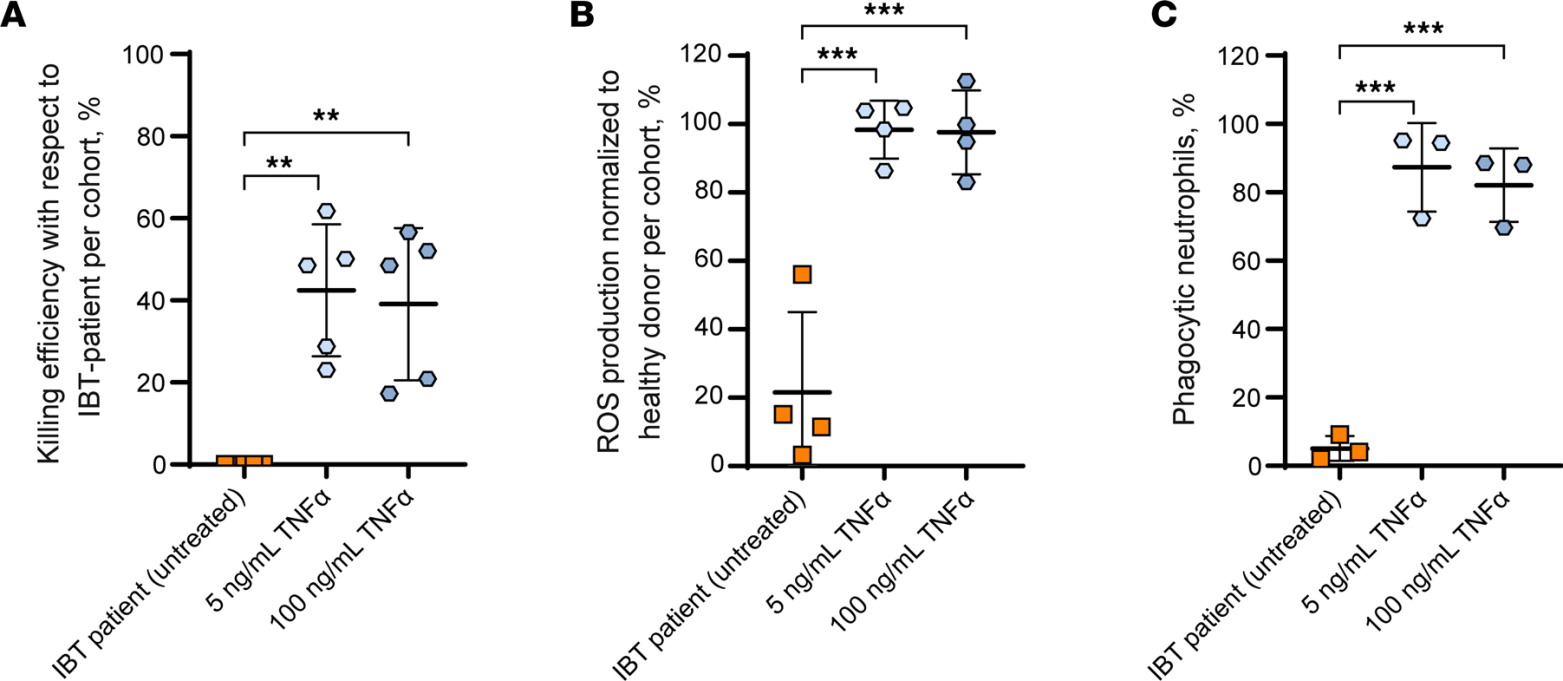
TNF-α compensated for immune defects against *A*. *fumigatus* in neutrophils from IBT-treated patients. Human neutrophils from IBT-treated patients or healthy donors were incubated for 4h with TNF-α and coincubated with *A*. *fumigatus* B5233 strain for all figure panels. (**A**) Neutrophils were incubated with *A*. *fumigatus* (MOI: 0.25) for 5h, and metabolic activity was estimated by resazurin-based assay. Data are shown as the percentage of *A*. *fumigatus* killing efficiency corresponding to neutrophils from each IBT-treated patient. Data are shown as mean ± SD, *n* = 5. (**B**) Neutrophils were incubated with 1 mg/mL *A*. *fumigatus* heat-killed hyphae. ROS production was measured by chemiluminescence using lucigenin. Data represent normalized ROS production from IBT-patient neutrophils to ROS production from healthy donors, per patient. Data are shown as mean ± SD, *n* = 4. (**C**) Neutrophils were coincubated with labeled *A*. *fumigatus* swollen spores (MOI: 10). The displayed percentage of phagocytic neutrophils (CD45-AF700^+^CD66b-APC^+^conidia-AF488^+^) was estimated based on the total number of viable neutrophils (CD45-AF700^+^CD66b-APC^+^). At minimum, 10,000 viable CD66b-APC^+^ events were recorded. Data represent the percentage of phagocytic neutrophils for neutrophils from each IBT-treated patient. Because of limits placed on peripheral blood draws for these patients, not all assays were performed on the 5 patients. Data are shown as mean ± SD, *n* = 3. **P < 0.01; ***P < 0.001.

Our data demonstrate that treating primary healthy human neutrophils with BTK inhibitors ex vivo potently affected neutrophil effector activity against *A*. *fumigatus*, a defect that exogenous TNF-α restored. However, whether this observation translated to patients on BTK inhibitors for the management of oncologic diagnosis remained unclear. Thus, we examined the restorative effect of TNF-α on the neutrophil immune response against *A*. *fumigatus* in patients actively treated with IBT. We isolated neutrophils from patients with B-lymphocyte leukemia undergoing IBT therapy. Patient or healthy donor neutrophils were treated with TNF-α for 3 hours, followed by stimulation with *A*. *fumigatus*. We then quantified pathogen killing, ROS production, and phagocytosis. Our results recapitulated our previous data: neutrophils from IBT-treated patients were less effective at responding to *A*. *fumigatus* when compared with neutrophils from healthy donors, but TNF-α rescued these defects to healthy control baseline (Figure 6 and Supplemental Figure 8). Together, these data demonstrate that BTK inhibitor–mediated neutrophil dysfunction can be reversed by TNF-α from patients on chronic IBT therapy.

## Discussion

Here, we unveiled the role of BTK inhibition on neutrophil antifungal effector functions. Specifically, we demonstrated that, even below typical plasma concentrations seen in chronically treated patients, BTK inhibitors caused significant immune defects in human neutrophils against the fungal pathogen. We identified TNF-α as one of the major pathways modified at a transcriptional level by BTK inhibition in neutrophils. Furthermore, we showed that exogenous TNF-α restores critical effector functions to contain and neutralize *A*. *fumigatus*. Importantly, these effects were not exclusive to healthy human neutrophils; they were also observed in neutrophils isolated from patients with B-lymphocyte leukemia receiving IBT treatment. Together, these data suggest that BTK functions as a master regulator of antifungal neutrophil activity.

Recognition of fungal cell wall components such as β-glucan and galactomannan by immune cells triggers antifungal immunity through phagocytosis, chemotaxis, production and release of proinflammatory cytokines, and ROS production (5). These pathways rely on the activation of tyrosine kinases, including BTK, to mediate immune effector functions to invading pathogens. Indeed, carbohydrate-like receptors (CLRs), integrins, TLRs, and the inflammasome are the primary activators of antifungal signaling cascades (54, 55). The integrin receptor CD11b/CD18 (Mac-1) and the CLR Dectin-1 are important receptors for β-glucan recognition in humans (56, 57) and participate in granulocyte activation, chemotaxis, cytotoxicity, and phagocytosis (58–61). We show that BTK does not abrogate Dectin-1 expression on IBT-treated neutrophils. The recognition of fungal hyphae or large clusters of conidia, potentially mediated by the same receptors, triggers neutrophil cooperation observed during swarming (39). Importantly, Mac-1 and Dectin-1 signals through kinases such as Syk, PI3K, and PKC (62), which in turn can modulates BTK activity. Interestingly, Mac-1 activation requires BTK in sterile inflammation (63). Activation of these pathways mediates the production of proinflammatory cytokines, phagocytosis of pathogens, and confinement of growing fungi inside neutrophil swarms (7). Although critical for antifungal immunity, these responses vary between immune cell types. In murine macrophages stimulated with *A*. *fumigatus*, TLR9/BTK/calcineurin/nuclear factor of activated T cells signaling cascade requires Dectin-1– and Syk-dependent phagocytosis, yet no changes in phagocytosis occur in response to inhibition of BTK (25, 64). Interestingly, in response to the fungal organism *Candida albicans* in macrophages, BTK localizes to the phagocytic cup and is necessary to generate mature phagosomal markers (9). Furthermore, BTK inhibition dampens phagocytic uptake. In the present study, we revealed the importance of functional BTK in mediating phagocytic uptake of *Aspergillus* conidia. While prior studies in macrophages suggest that phagocytosis of *A*. *fumigatus* remains similar in the presence and absence of BTK inhibition (63), it is possible that immortalized cell lines and primary human neutrophils respond differently. These data suggest that that role of BTK in phagocytosis may be species and immune cell specific. The precise mechanism of BTK modulation of phagocytosis in neutrophils remains unknown.

Neutrophil ROS production facilitates fungal killing. Inadequate production of ROS enables fungal pathogens to invade host tissues. Individuals with deficiencies in key components of ROS production, such as subunits of the NADPH oxidase complex, are at risk of recurrent and severe fungal infections (65, 66), highlighting the importance of ROS in containing fungal infections. Given the reduced phagocytic capacity of neutrophils treated with BTK inhibitors, we would expect reduced downstream ROS production. Indeed, our data suggest that BTK inhibition only impairs phagocytosis-dependent intracellular and extracellular ROS production in response to *A*. *fumigatus*. These data confirm previous studies that demonstrate dampened ROS production in neutrophils isolated from patients with 1 month and 3 months of IBT therapy (18). Since most of our investigations utilized neutrophils isolated from healthy volunteers, these results suggest that the reduction of effector functions against *A*. *fumigatus* is triggered by the BTK inhibition rather than the underlying disease requiring treatment with BTK inhibitors (e.g., chronic lymphocytic leukemia, graft-versus-host disease). Overall, we argue that BTK regulates neutrophil phagocytosis, a fundamental step in the recognition of fungal pathogens, which subsequently leads to ROS production and, ultimately, the killing of the pathogen.

Cases of aspergillosis dominate the invasive fungal infections in patients receiving BTK inhibitory therapy compared with other fungal pathogens. Interestingly, there is a proclivity of disseminated *Aspergillus* infection to the CNS in patients treated with a BTK inhibitor, with 40%–60% of IBT-associated aspergillosis presenting cerebrally (20, 43, 67, 68). The mechanisms underpinning the susceptibility of the CNS to invasive aspergillosis remains unknown. While the role of BTK inhibition in innate immune cells in the periphery has been demonstrated by data presented here and in other studies, the role of BTK inhibition on resident immune cells (i.e., microglia and astrocytes) in the brain, or the blood-brain barrier function in the setting of fungal infection, remains unknown. BTK inhibition dampens microglial and astrocyte LPS-induced activation and proinflammatory cytokine production, including TNF-α (69). Here, we suggest that neutrophil dysfunction is important to BTK inhibition–associated aspergillosis. In a model of cerebral aspergillosis, no change in the number of neutrophil or γδ T cells were observed, although other immune cells were drastically lower (70). Neutrophils produce low levels of TNF-α compared with other inflammatory cells, such as macrophages, DCs, NK cells, and T cells (71). Perhaps these cells compensate for the decreased TNF-α produced by neutrophils during treatment with BTK inhibitors, resulting in less established fungal infections in the periphery. Given the immunomodulatory role of IBT in a murine model and the fact that microglia, astrocytes, and neurons are the primary source of TNF-α in the CNS (69, 72), it is possible that low TNF-α secretion cannot be compensated in the brain, enabling fungal organisms to establish an infection in patients receiving BTK inhibition. Further investigations on the role of local and recruited immune cells in BTK inhibition–associated CNS aspergillosis are warranted.

Given the propensity of invasive fungal infections in patients treated with BTK inhibitors, we examined opportunities to bypass BTK inhibition and restore neutrophil effector functions. Our transcriptional analyses highlight an upregulation of numerous components in the TNF-α signaling pathway, including the receptor. Interestingly, we reveal a downregulation of TNF-α itself in BTK-inhibited neutrophils. In concordance with these observations, BTK inhibition impairs TNF-α production in monocyte-derived macrophages, alveolar macrophages, and γδ cells in response to *A*. *fumigatus*, *Streptococcus pneumoniae*, and *Mycobacterium tuberculosis* (25, 26, 28, 64). Since TNF-α can modulate neutrophil recruitment, an insufficient production of TNF-α by macrophages and γδ cells in response to *Aspergillus* may contribute to blunted neutrophil recruitment and host defense in patients treated with BTK inhibitors.

Since TNF-α was downregulated, we hypothesized that exogenous TNF-α could restore neutrophil effector function, despite other pathway components remaining available. Upon exposure to exogenous TNF-α, BTK-treated neutrophils recovered effector activity. While these results are encouraging, the use of TNF-α during fungal infections in patients treated with a BTK inhibitor is not feasible, given the numerous off-target effects and induction of severe endotoxic shock. TNF-α is an essential proinflammatory cytokine, but under certain circumstances, too much TNF-α indirectly induces cell death through amplified proinflammatory response (73). Due to exacerbated inflammation, anti–TNF-α biologics are approved for autoimmune diseases such as rheumatoid arthritis, psoriasis, Crohn’s disease, and ulcerative colitis (74). These TNF antibody treatments carry an increased risk of fungal infection, particularly in those treated for gastrointestinal disease (75, 76). Thus, understanding how exogenous TNF-α exerts protective effects may expand beyond BTK inhibitor treatments to include high-risk patients on TNF biologics. Further studies are warranted to identify downstream targets with better therapeutic potential in these patients.

Here, we reveal that stimulation of the TNF-α signaling pathway compensates for defects in neutrophils chronically exposed to IBT. GM-CSF can also compensate for these defects (41), while IFN-γ, G-CSF, IL-1β, and IL-8 were unable to do so. Notably, both GM-CSF and TNF-α converge on the PI3K/AKT pathway, and this convergence may provide insight into the specificity of this response. A small molecule that activates this pathway may be another approach to overcome the effects of BTK inhibition. Thus, further research will seek to understand better the specific effectors downstream of TNF-α supplementation responsible for the rescue of neutrophil defects induced by BTK inhibitor treatments to enable more targeted therapies. Overall, the results presented here significantly enhance our insights into the immunomodulatory properties of BTK inhibition and identify pathways that may be leveraged to improve patient outcomes.

## Methods

### Sex as a biological variable.

Neutrophils were isolated from both men and women. No differences in were observed between these groups. All data shown in this manuscript represent pooled samples from neutrophils isolated from both men and women in the given treatment group.

### Strains and culture conditions.

*A*. *fumigatus* strains B5233 (77), Af293 (78), ATCC46645 (79), and CEA10 (80, 81) were grown in glucose minimum media (GMM) (82) agar at 37°C for 3 days. Conidia were harvested using sterile water with 0.01% Tween 20 (Sigma-Aldrich, P9416) and purified using a 40 μm cell strainer (CELLTREAT, 229481). Spores were washed 3 times with sterile PBS (Corning, 21-040-CM) and counted on a LUNA automated cell counter (Logos Biosystems). Swollen conidia were obtained by incubating *A*. *fumigatus* conidia in cRPMI media (RPMI-1640 [Corning, 10-040-V] supplemented with 9% FBS [Invitrogen, 26140079], 158 μM penicillin, 152 μM streptomycin [both from Gibco, 151410122], 1.8 mM L-glutamine [Gibco, 25030081], 9 mM HEPES [Gibco, 15630080], 63.3 μM β-mercaptoethanol [Sigma-Aldrich, M6250]) in the presence of 0.5 mg/mL voriconazole (VRZ; Sigma-Aldrich, PZ0005-25MG) for 6 hours at 30°C with agitation. Swollen conidia were centrifuged for 3 minutes at 16,000*g*, washed with sterile PBS 3 times, and resuspended in cRPMI.

Heat-killed *A*. *fumigatus* was grown as previously described (83). Briefly, 3 × 10^7^ CFUs were inoculated in 5 mL of YPD media (yeast extract [Bacto, 212750], peptone [Bacto, 211677], dextrose [Sigma-Aldrich, D9434]) and grown at 37°C overnight to generate hyphae. Mycelium was carefully collected, centrifuged for 3 minutes at 16,000*g*, washed with sterile PBS 3 times, weighted, and resuspended in 1 mL of PBS. Hyphae was heat-killed using three 95°C cycles of 10 minutes each, vortexing between cycles. Heat-killed hyphae was grinded using sterile 1.5 mL pestles (Bio Plas Inc., 4030-PB). Grounded heat-killed hyphal elements were washed 3 times in PBS and resuspended to 1 mg of material per mL and stored at 4°C.

### Human neutrophil isolation.

Peripheral blood from 18 healthy volunteers and 5 patients withB-lymphocyte leukemia treated with IBT were collected in K2 EDTA-treated tubes (BD Biosciences, 367899) and centrifuged at 1,500*g* for 15 minutes at room temperature. Neutrophils were isolated from the buffy coat by negative isolation using the EasySep Direct Human Neutrophil Isolation Kit (Stemcell Technologies Inc., 19666), according to the manufacturer’s instructions. Isolated neutrophils were resuspended in cRPMI, assessed viability using Acridine Orange/Propidium Iodide (New England BioGroup, F23001), and analyzed by flow cytometry to confirm purity using a BD FACSCelesta Cell Analyzer and the Diva software (BD Biosciences). All data shown are representative of at least 3 independent experiments using different donors.

### Drugs, cytokines, and monoclonal antibody treatments.

Unless stated otherwise, neutrophils were incubated with ABT, IBT, ZBT (Cayman Chemical, 19899, 16274, and 28924, respectively), or the solvent vehicle control (0.1% DMSO, Sigma-Aldrich, D2650) at the indicated concentrations for 4 hours at 37°C and 5% CO_2_. When necessary, a 4-hour cytokine treatment started 30 minutes after adding the BTK inhibitor. The following cytokines and their doses were used: 5 ng/mL and 100 ng/mL TNF-α (Invivogen, rcyc-htnfa); and 100 ng/mL IFN-γ (BioLegend, 570206); 100 ng/mL IL-1β, 50 ng/mL IL-8, or 100 ng/mL G-CSF (PeproTech, 200-01B, 200-08M, 315-02, respectively).

For blocking of phagocytosis, 20 μM of cytochalasin D (Sigma-Aldrich, C8273-1MG) was used prior to adding any treatment. For TNF-α blocking experiments, 25 μg/mL IFM (MGH Pharmacy) was added 15 minutes before adding TNF-α. For the TNF-α time-course experiment, neutrophils were treated with 5 ng/mL TNF-α for 0-, 15-, or 30-minute stimulation with *A*. *fumigatus*. For transcription inhibition experiments, 1 μg/mL actD (Sigma-Aldrich, A1410-2MG) was used 30 minutes after adding IBT. Cytokines were added 15 minutes after actD.

### Aspergillus metabolic assay (neutrophil killing assay).

Neutrophils were treated with DMSO or either ABT, IBT, or ZBT as described above. Unless stated otherwise, 200,000 neutrophils/well were stimulated with 50,000 *A*. *fumigatus* swollen conidia in Falcon 96-well plates (Corning, 353219). VRZ was used at 16 μg/mL as a control for suppression of *A*. *fumigatus* metabolic activity. After 5 hours, neutrophils were lysed using NP-40 lysis buffer (75 mM NaCl [Fisher BioReagents, 358212], 2.5 mM MgCl_2_·6H_2_O [Sigma-Aldrich, M2393], 0.5% NP-40 [Fluka Chemi, 74385] [pH 7.5]) for 5 minutes on ice. Media were then supplemented with MOPS-cRPMI (cRPMI containing 165 mM MOPS [Fisher BioReagents, 308500], 2% glucose [Sigma-Aldrich, G5767] [pH 7.0]) and 1:10 PrestoBlue (Invitrogen, A13261), and conidia were allowed to germinate for 12.5 hours at 37°C. Thereafter, fluorescence (560/590 nm) was recorded every 30 minutes for 24 hours. *A*. *fumigatus* metabolic activity was determined by resorufin fluorescence using an SpectraMax i3x microplate reader (Molecular Devices). *A*. *fumigatus* killing was estimated using the Gompertz function as described:


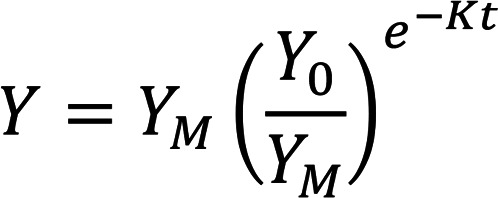
  (Equation 1)

Where *Y_0_* is the starting metabolic activity, *Y_M_* is the maximum metabolic activity, and *K* describes the metabolic rate; equivalently, 1/*K* describes the delay (inflection point). We estimated *A*. *fumigatus* killing by finding the ratio of of a neutrophil and *A*. *fumigatus* treatment with respect to the of an *A*. *fumigatus* control condition (i.e., spores only, IBT treatment) as described:


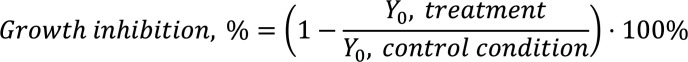
 (Equation 2)

For all figures, the data are presented as the percent of growth inhibition after performing the linear regression analysis using Gompertz fit with 95% CI (with the exception of Figure 1, A and B, which shows the raw data used to calculate the growth inhibition in Figure 1A).

### Neutrophil extracellular ROS production.

Using 96-well plates (Greiner Bio-One, 655083), 100,000 neutrophils in cRPMI were stimulated for 4 hours at 37°C with 1 mg/mL *A*. *fumigatus* heat-killed hyphae, 1 μg/mL phorbol 12-myristate 13-acetate (PMA; Stemcell Technologies Inc., 74042), or β-glucan-coated beads (84) at 5:1 bead/neutrophil ratio in the presence of 0.15 μM lucigenin (bis-N-methylacridinium nitrate; Enzo Life Sciences Inc., ENZ-52154) (85, 86). Extracellular ROS–dependent chemiluminescence (87) was measured every 5 minutes for 4 hours using an SpectraMax i3x microplate reader.

### Flow cytometry (conidial phagocytosis, Dectin-1 expression, and intracellular ROS).

For conidial phagocytosis, *A*. *fumigatus* swollen conidia were labeled using 20 μg/mL AF488-NHS ester (succinimidyl ester) (Invitrogen, A20000) in PBS for 1 hour with agitation, rinsed with PBS, and resuspended in FACS buffer (PBS, 2% FBS, 1 mM EDTA [Sigma-Aldrich, E7889]). In total, 200,000 neutrophils in cRPMI were stimulated with AF488-labeled *A*. *fumigatus* at multiplicity of infection (MOI) 10:1, in a 96-well V-bottom nontreated polypropylene microplate (Corning, 3357) for 2 hours at 37°C and 5% CO_2_. For Dectin-1 expression, 1 × 10^6^ neutrophils in cRPMI were incubated with either solvent control (DMSO) or various concentrations of IBT for 4 hours at 37°C and 5% CO_2_. For intracellular ROS production, 1 × 10^6^ neutrophils in cRPMI were incubated in conical tubes with either DMSO, various concentrations of IBT, or media alone for 4 hours at 37°C and 5% CO_2_. Neutrophils were then moved to FACS tubes, and 1 μM dihydroethidium (DHR, Invitrogen, D11347) was added and then stimulated with 1 mg/mL heat-killed *A*. *fumigatus* hyphae (B5233 strain), 5 ng/mL PMA, or media alone for 1 hour at 37°C and 5% CO_2_. After stimulation in all experiments, cells were incubated on ice for 10 minutes. Cells were washed with FACS buffer and treated with Human TruStain FcX, 7-AAD (Biolegend, 422302) (viability) for phagocytosis and Dectin-1 studies, anti–CD66b-APC, anti–CD45-AF700, and/or anti–Dectin-1-PE (BioLegend, 422302, 305118, 304024, and 355404, respectively). Experimental samples were analyzed using a BD FACSCelesta Cell Analyzer (minimum 10,000 viable CD66b^+^ events) and the BD FACSDiva software, v.10. The gating strategy is outlined in Supplemental Figure 9.

### Neutrophil swarming assay.

A microarray printing platform (Picospotter PolyPico) was used to print a solution of 0.1% poly-L-lysine (Sigma-Aldrich, P8920) and ZETAG 8185 targets (BASF) with 100 μm diameter in 8 × 8 arrays on a 16-well format on ultraclean glass slides (Thermo Fisher Scientific) (36). Slides were screened by microscopy for printing accuracy, dried at room temperature for 2 hours, and assembled into 16 chambers using ProPlate Multi-Well Chambers (Grace Bio-Labs, 204860). Wells were loaded with 50 μL of *A*. *fumigatus* resting conidia in sterile H_2_O, incubated for 10 minutes with agitation, and thoroughly washed with PBS to remove unbound conidia. Wells were screened by microscopy to ensure appropriate patterning of targets onto the spots. *A. fumigatus*-seeded targets were located using the Nikon Perfect Focus system and multipoint function. Wells were loaded with 500,000 neutrophils stained with 4 μM Hoechst (Thermo Fisher Scientific, H3570) in 200 μL of swarming media (Iscove’s Modified Dulbecco’s Media [Cytivia Life Sciences, SH30228.01] with 20% FBS). When using chemical inhibitors and cytokines, neutrophils were preincubated as described above in swarming media. Live-cell imaging was conducted using a Nikon Ti-E inverted microscope. An excitation light source, 4-W laser (Coherent), was used to produce excitation wavelengths of 405 and 488 nm using an acoustic optical tunable tuner. To acquire differential interference contrast images, a polarizer (MEN 51941; Nikon) and Wollaston prisms (MBH76190; Nikon) were used. Images were collected using a 10× objective and an EM-CCD camera (C9100-13; Hamamatsu). Image acquisition was performed using MetaMorph 7.10 (Molecular Devices). Image analysis was performed using Fiji (88) as described by Hopke et al. (36, 89), and raw image data files were processed using Adobe Photoshop 2023.

### RNA extraction and qPCR.

In total, 400,000 neutrophils were incubated at 37°C and 5% CO_2_ in the presence or absence of *A*. *fumigatus* (MOI: 2.5). After 6 hours, cells were centrifuged for 5 minutes at 500*g* and supernatants were removed. Cell pellets were resuspended in 350 μL of Buffer RLT (Qiagen, 79216) containing 1% β-mercaptoethanol and incubated on ice for 10 minutes. Lysates were homogenized using QIAshredder columns (Qiagen, 79656). Homogenized lysates were mixed with RNase-free 70% ethanol and purified using the RNeasy Mini Kit (Qiagen, 74134) according to the manufacturer’s instructions. RNA concentrations were measured using a NanoDrop One (Thermo Fisher Scientific, ND-ONE-W), and 1% agarose gels were used to verify RNA integrity.

RNA samples were treated with ezDNase enzyme (Invitrogen). For cDNA synthesis, 15 ng of RNA were combined with the SuperScript IV VILO Master Mix kit (Invitrogen) according to the manufacturer instructions. Reverse transcription was performed for 10 minutes at 50°C. mRNA was quantified for *CXCL8* (TaqMan Gene Expression Assays, Hs00174103_m1) and the housekeeping gene *GAPDH* (TaqMan Gene Expression Assays, Hs02758991_g1) by quantitative PCR (qPCR) using TaqMan Fast Advanced Master Mix (Applied Biosystems, 4444557) using 2 μL of cDNA in 20 μL reactions, with 40 cycles of 3 seconds at 95°C followed by 30 seconds at 60°C (Applied Biosystems 7500 Fast Real-Time PCR). Transcript levels were normalized using *GAPDH*.

### NanoString nCounter analysis.

Transcriptional profiling was obtained using the nCounter Human Host Response panel (NanoString Technologies, Q-21898) according to the manufacturer’s instructions. Briefly, 25 ng of total RNA were used for hybridization reactions at 65°C for 22 hours, loaded onto a Sprint cartridge, and analyzed using an nCounter SPRINT Profiler (NanoString Technologies). Data analysis was performed using nSolver 4.0. To adjust for differences in total RNA per lane, hybridization efficiency, and posthybridization processing, the counts of 773 target RNAs were normalized based on negative controls (background subtraction) and the geometric mean of 12 positive control RNA counts.

### ELISA.

In total, 2,000,000 neutrophils were treated for 4 hours with 0.3 μM IBT or DMSO and incubated for 5 hours at 37°C and 5% CO_2_ in the presence or absence of *A*. *fumigatus* (MOI: 2.5). TNF-α from the supernatant was measured using the ELISA MAX Deluxe Set (BioLegend, 430204) following the manufacturer’s instructions.

### Statistics.

Statistical analysis was performed using GraphPad Prism 9 software for all studies except for NanoString studies, which was performed using nSolver Advance Analysis 2.0. Data are presented as mean ± SD or percentage ± 95% CI. For extracellular ROS production studies, the AUC was calculated. For all studies except for NanoString experiments, statistical differences were obtained using an ordinary 1-way ANOVA and Tukey’s multiple-comparisons test with a single pooled variance. *P* ≤ 0.05 was considered significant. For NanoSting studies, the fold changes, *P* values, and adjusted *P* values were obtained using the Benjamini-Yekutieli method. Only genes with an adjusted *P* ≤ 0.05 and a log_2_ fold-change of ± 1.5 were significant.

### Study approval.

The use of human blood samples to isolate primary neutrophils was approved by the IRB at Massachusetts General Hospital (protocol no. 2015P000818). Informed consent for data used was provided by all participants prior to participation in the study.

### Data availability.

NanoString raw data files and normalized data are available through the GEO database (accension no. GSE264298). Raw data for figures presented in this manuscript are available in the Supporting Data Values file.

## Author contributions

DAVB, OWH, AH, and JMV conceptualized the study and developed the methodology; DAVB, OWH, KJB, PS, AJC, KDT, AH, HEH, KNJ, DJF, JLR, and CR performed experiments; DAVB, OWH, KJB, AH, SRV, and JMV, analyzed and interpreted data; DAVB, KJB, CR, RAW, JSA, and JMV coordinated and managed experiments using clinical samples; DAVB, OWH, RAW, and JMV drafted the paper; DAVB, OWH, KJB, PS, AJC, KDT, AH, HEH, SRV, KNJ, DJF, JLR, CR, MKM, RAW, DI, JSA, and JMV reviewed and edited the paper. DAVB performed most of the experiments and is listed as the first author of the co–first authors.

## Supplementary Material

Supplemental data

Supporting data values

## Figures and Tables

**Table 1:**
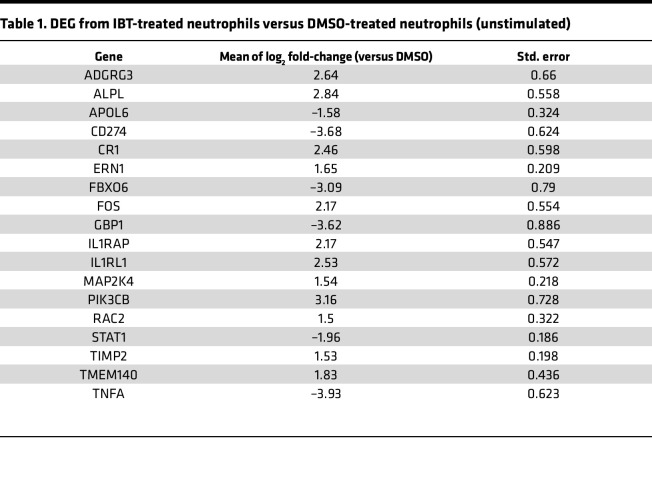
DEG from IBT-treated neutrophils versus DMSO-treated neutrophils (unstimulated)

## Version 1 05/07/2024

In-Press Preview

## Version 2 06/24/2024

Electronic publication
