# Supplementary material for: BTK inhibitor–induced defects in human neutrophil effector activity against Aspergillus fumigatus are restored by TNF-α
Source: JCI Insight. 2024 May 7;9(12):e176162. doi: 10.1172/jci.insight.176162 (PMC11383172; doi:10.1172/jci.insight.176162)
Supplement: Supplemental data [file jciinsight-9-176162-s133.pdf]

SUPPLEMENTAL FIGURES

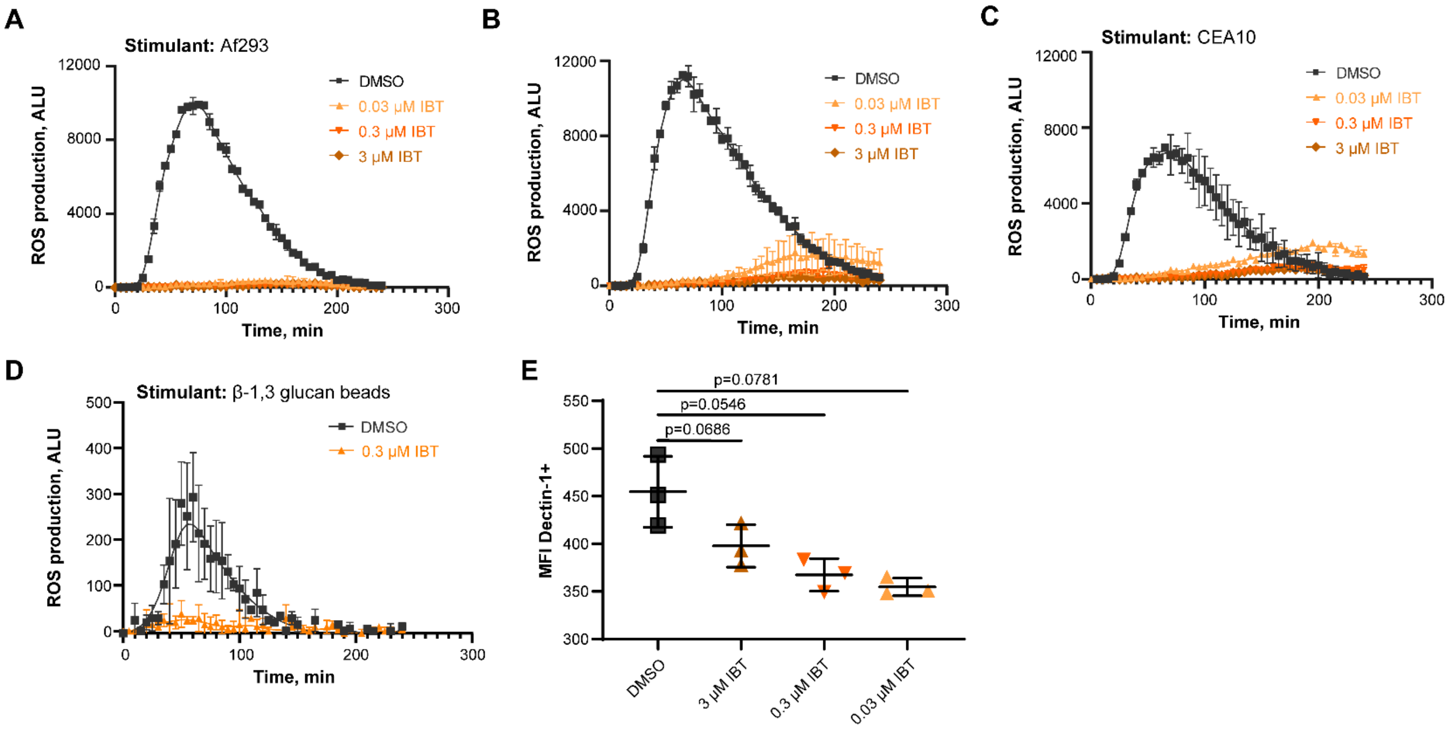

Supplemental Figure 1. IBT impaired neutrophil ROS production against different *A. fumigatus* strains and β-glucan-coated beads. Neutrophils were treated with different concentrations of IBT for 4h prior stimulation with 1 mg/mL of heat-killed hyphae from *A. fumigatus* Af293 **(A)**, *A. fumigatus* CEA10 **(B)**, *A. fumigatus* ATCC 46645 **(C)**, or β-glucan-coated beads at a 5:1 bead-to-neutrophil ratio **(D)**. ROS production was measured by chemiluminescence using lucigenin. **(E)** Dectin-1 expression in human neutrophils treated with IBT or DMSO for 4h as measured by flow cytometry (CD11b+/CD66b^{+}/Dectin-1^{+}). One-way ANOVA with Bonferroni's multiple comparisons test. Error bars are SD, n = 3, data representative from at least three independent experiments.

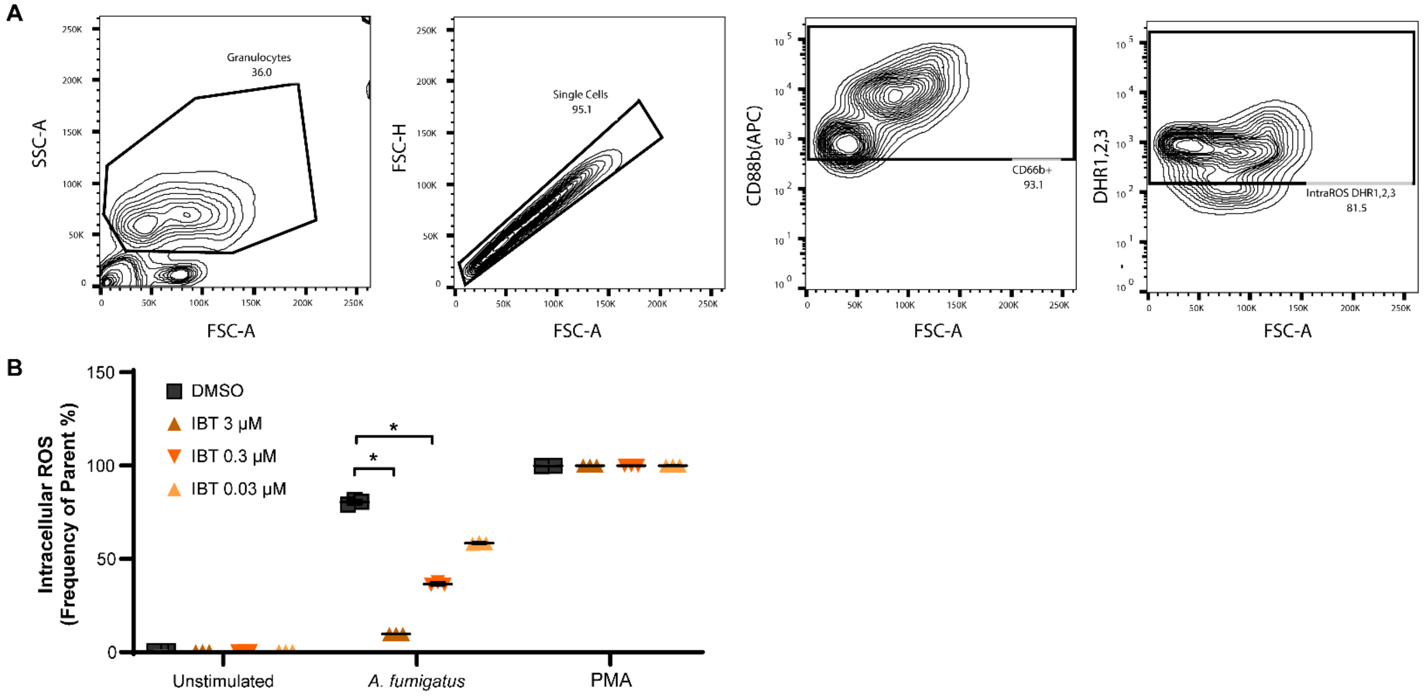

Supplemental Figure 2. Intracellular ROS production in response to *A. fumigatus* dampened by IBT treatments. Human neutrophils were pre-treated with IBT at varying concentrations or DMSO for 4h and incubated with *A. fumigatus* B5233 strain heat-killed hyphae (1 mg/mL) or PMA (5 ng/mL) for 1h. The displayed percentage of intracellular ROS production in neutrophils ($CD66b^+$/ DHR^+) was calculated based on the total number of viable neutrophils ($CD66b^+$). A minimum of 10,000 viable $CD66b^+$ events were recorded. Two-way ANOVA with Bonferroni's multiple comparisons test; *p<0.05. Error bars are SD, *n* = 3.

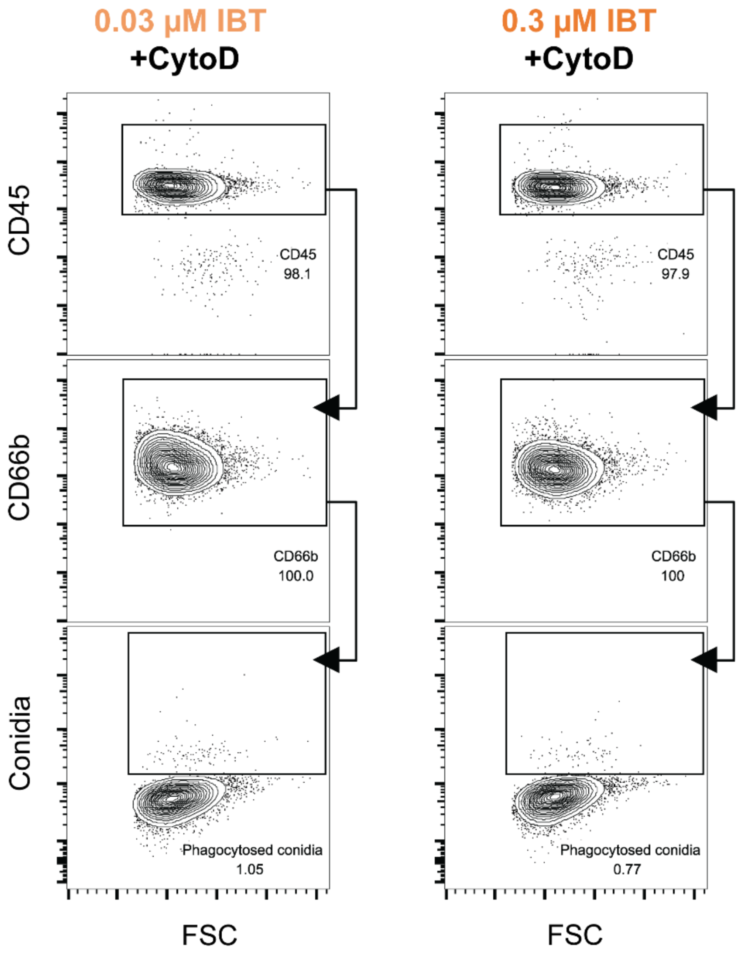

Supplemental Figure 3. Cytochalasin D inhibited phagocytosis at similar levels across IBT treatments. Human neutrophils were pre-treated with cytochalasin D (CytoD), treated with IBT for 4h, and incubated with fluorescently labeled *A. fumigatus* B5233 strain swollen spores (MOI: 10). The displayed percentage of phagocytic neutrophils ($CD45^+/CD66b^+/$ $conidia^+$) was calculated based on the total number of viable neutrophils ($CD45^+/CD66b^+$). A minimum of 10,000 viable $CD66b^+$ events were recorded.

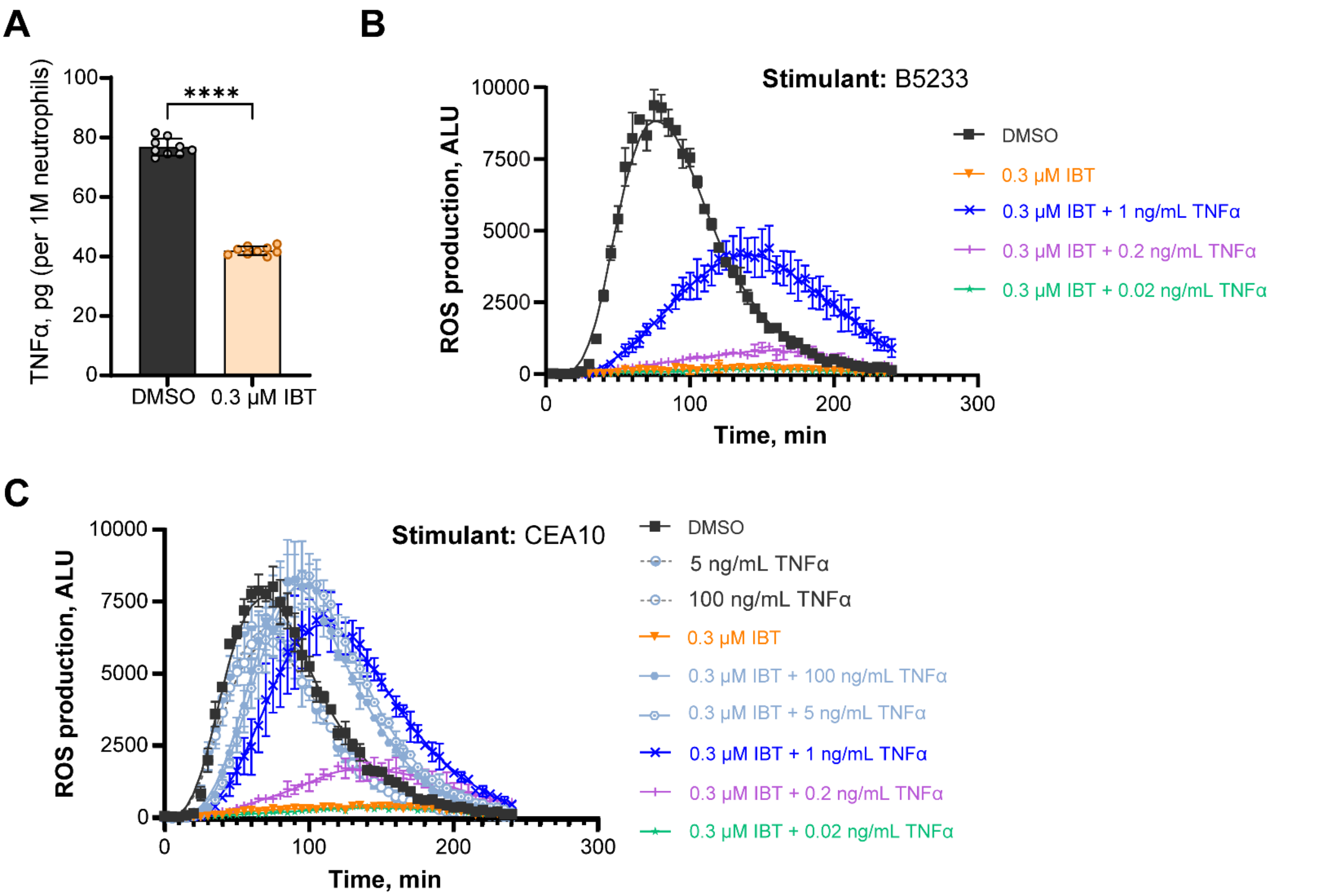

Supplemental Figure 4. TNFα compensated for ROS production in IBT-treated neutrophils in a dose-dependent manner. (A) ELISA for soluble TNFα from the supernatants of neutrophils treated for 4h with 0.3 µM IBT or DMSO and stimulated with *A. fumigatus* B5233 strain (MOI:2.5) for 5h. Error bars are SD, $n = 9$, data representative from at least three independent experiments. Two-tailed, unpaired t-test; **** $p < 0.0001$. **(B, C)** Neutrophils were treated with 0.3 µM IBT for 30 min followed by 4h TNFα at different concentrations prior to stimulation with 1 mg/mL heat-killed hyphae from *A. fumigatus* strains B5233 (B) or CEA10 (C). ROS production was measured via chemiluminescence using lucigenin. Error bars are SD, $n = 3$.

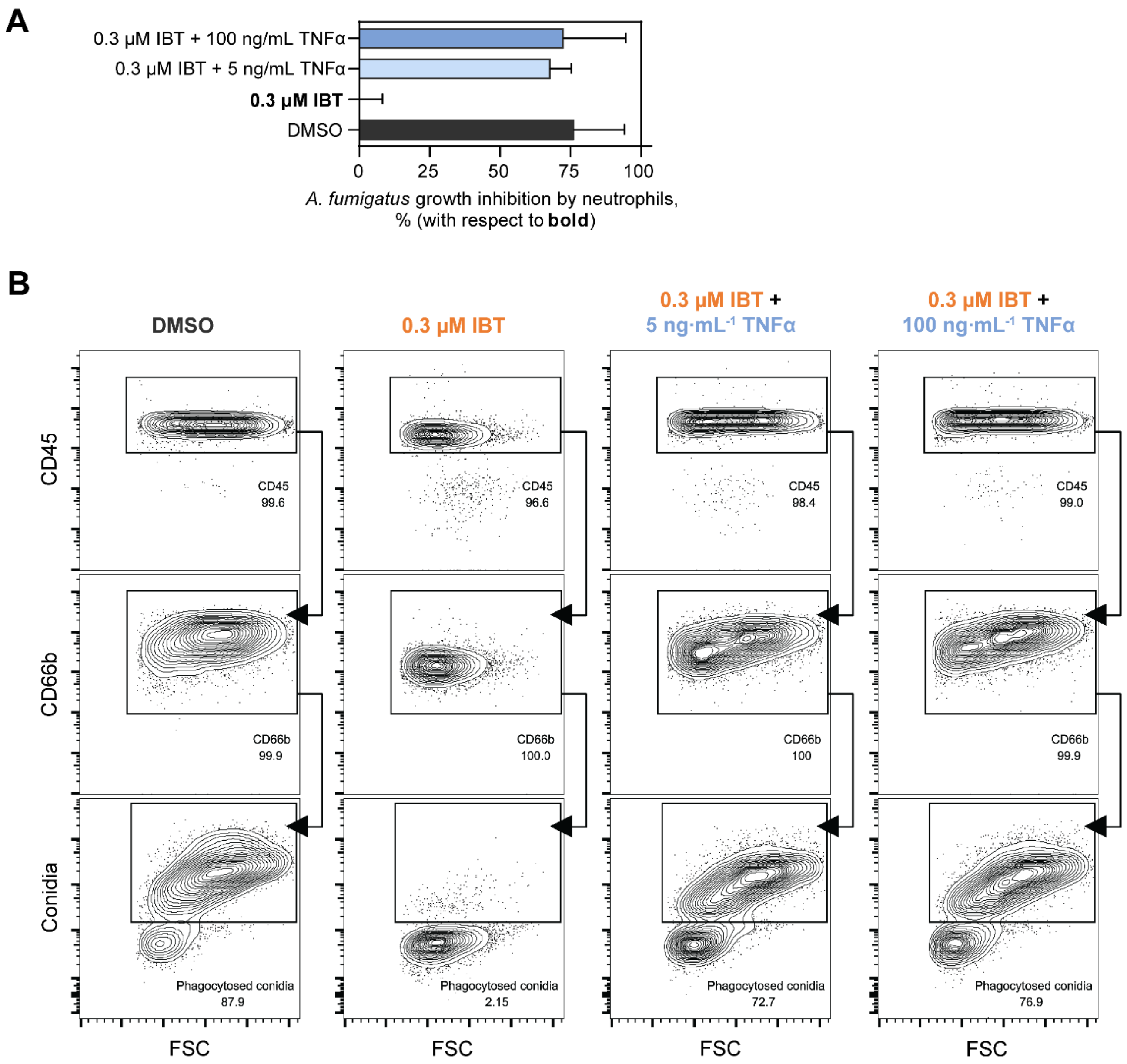

Supplemental Figure 5. IBT impaired neutrophil activity against *A. fumigatus* strain CEA10. Human neutrophils were treated with 0.03 µM IBT, 0.3 µM IBT, or DMSO for 30 min, followed by a 4h incubation with TNFα at the indicated concentrations and co-incubated with *A. fumigatus* CEA10 strain for all figure panels. All data are representative of at least three independent experiments. **(A)** Neutrophils were co-incubated with *A. fumigatus* (MOI:0.25) for 5h, and metabolic activity was measured by resazurin-based assay. Data calculated through time course study (see raw data in Supplemental Materials) and panel represents the output from linear regression analysis using Gompertz fit with percentages of growth inhibition of *A. fumigatus* by neutrophils in reference to IBT-treated neutrophils. Error bars are 95% CI, $n = 3$. **(B)** Neutrophils were co-incubated with labeled *A. fumigatus* swollen spores (MOI: 10). The displayed percentage of phagocytic neutrophils ($CD45^+/CD66b^+/conidia^+$) was estimated based on the total number of viable neutrophils ($CD45^+/CD66b^+$). A minimum of 10,000 viable $CD66b^+$ events were recorded.

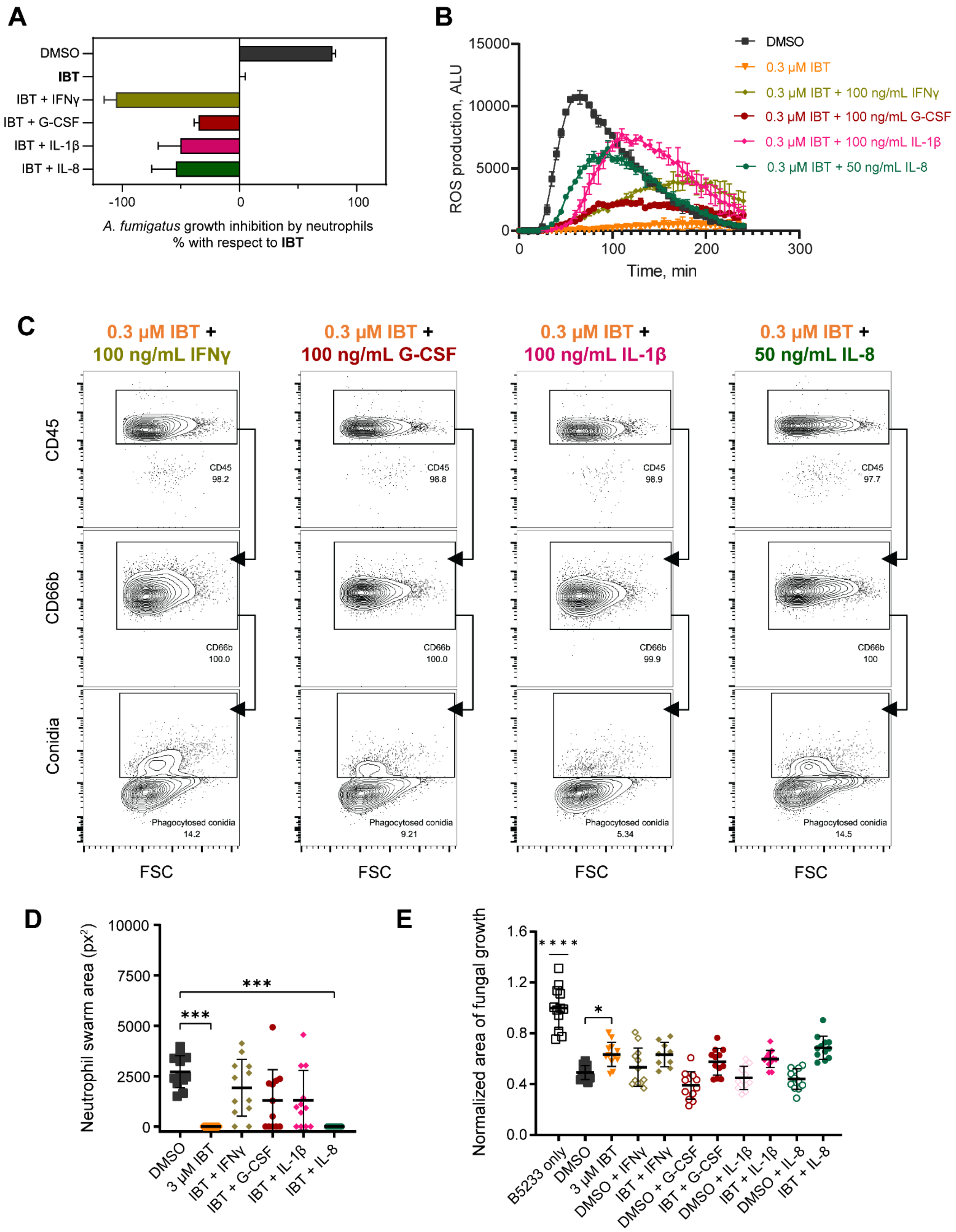

Supplemental Figure 6. G-CSF, IL-1β, IL-8, and IFNγ were unable to compensate for IBT-induced defects in neutrophil activity against *A. fumigatus*. Human neutrophils were treated with 0.3 µM IBT or DMSO for 30

min followed by a 4h incubation with 100 ng/mL G-CSF, 100 ng/mL IL-1β, 100 ng/mL IFNγ, or 50 ng/mL IL-8 and co-incubated with *A. fumigatus* B5233 strain for all figure panels. All data are representative of at least three independent experiments. **(A)** Neutrophils were incubated with *A. fumigatus* (MOI:0.25) for 5h, and metabolic activity was measured by resazurin-based assay. Data calculated through time course study (see raw data in Supplemental Materials) and panel represents the output from linear regression analysis using Gompertz fit with percentages of growth inhibition of *A. fumigatus* by neutrophils in reference to IBT-treated neutrophils. Error bars are 95% CI, $n = 3$. Two-tailed, unpaired t-test IBT alone vs IBT + cytokine; $p = 0.0007$ for G-CSF, $p = 0.0106$ for IL-1β, $p = 0.0111$ for IL-8, $p < 0.0001$ for IFNγ. **(B)** Neutrophils were stimulated with 1 mg/mL *A. fumigatus* heat-killed hyphae. ROS production was measured by chemiluminescence using lucigenin. Error bars are SD, $n = 3$. **(C)** Neutrophils were co-incubated with labeled *A. fumigatus* swollen spores (MOI: 10). The displayed percentage of phagocytic neutrophils ($CD45^+/CD66b^+/conidia^+$) was estimated based on the total number of viable neutrophils ($CD45^+/CD66b^+$). A minimum of 10,000 viable $CD66b^+$ events were recorded. **(D)** Area of neutrophil swarm after 200 min. **(E)** Area of fungal growth normalized to the growth of *A. fumigatus* without neutrophils after 16h. Error bars for C-D are SD, $n = 12$, data representative from at least three independent experiments. Ordinary one-way ANOVA and Tukey's multiple comparisons test with a single pooled variance, * $p < 0.05$; *** $p < 0.001$.

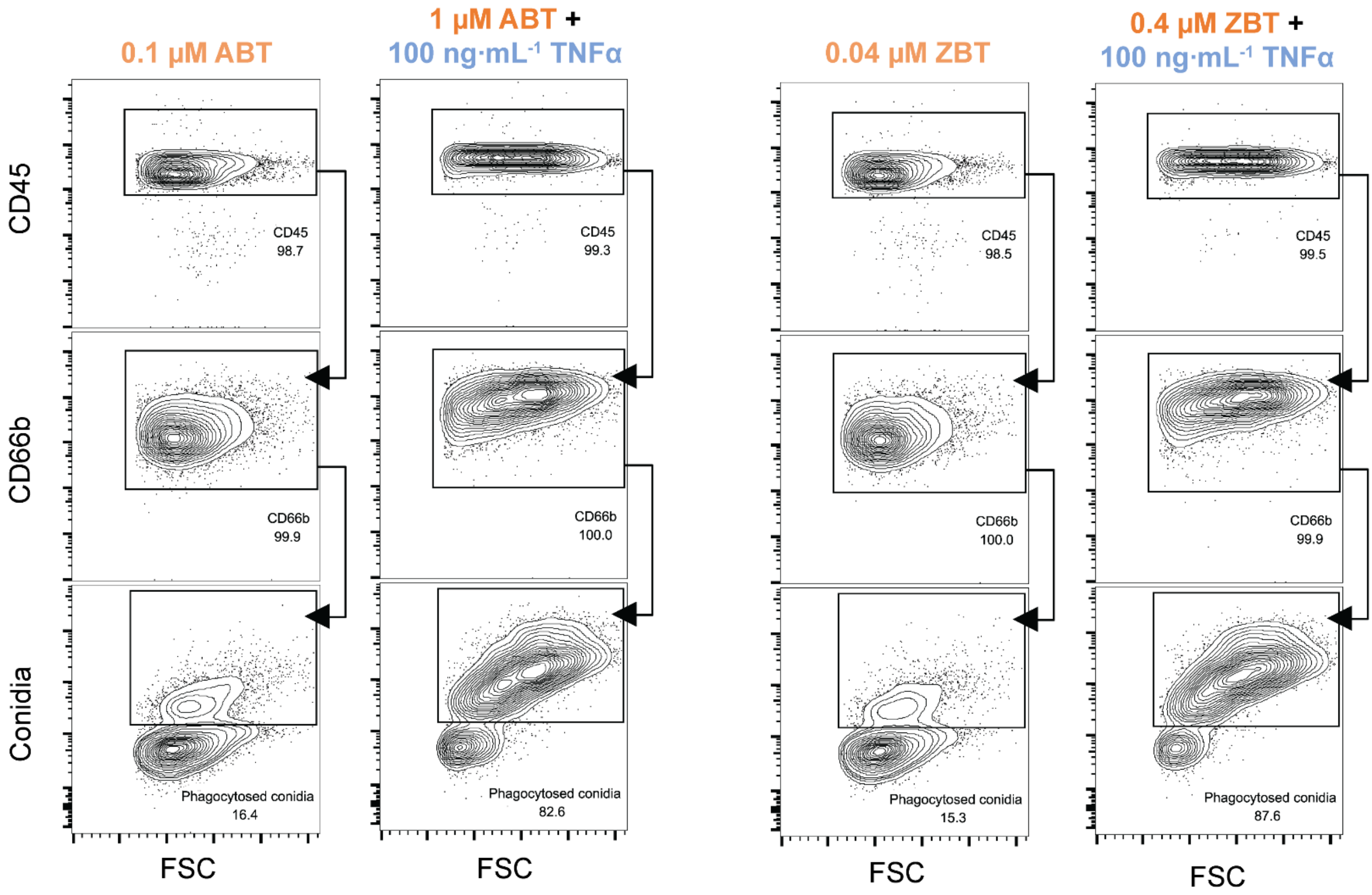

Supplemental Figure 7. TNFα restored defects caused by multiple BTK inhibitors on neutrophil immune activity against *A. fumigatus* Human neutrophils were treated with ABT or ZBT for 30 min followed by a 4h incubation with TNFα and co-incubated with *A. fumigatus* B5233 strain. For all panels, data are representative of at least three independent experiments. Neutrophils treated with ABT or ZBT were co-incubated with labeled *A. fumigatus* swollen spores (MOI: 10). The displayed percentage of phagocytic neutrophils ($CD45^+$/$CD66b^+$/$conidia^+$) was calculated based on the total number of viable neutrophils ($CD45^+$/$CD66b^+$). A minimum of 10,000 viable $CD66b^+$ events were recorded.

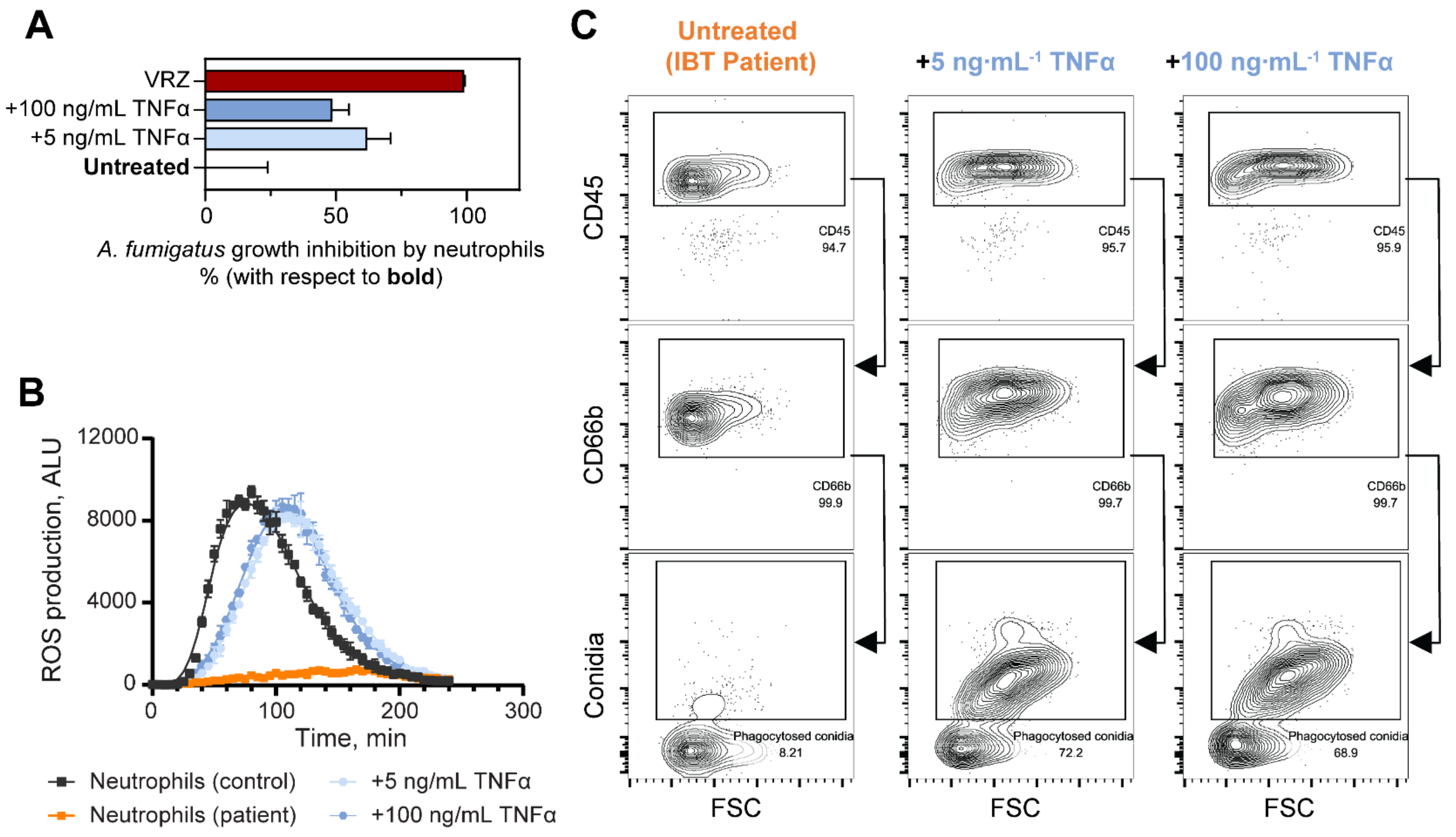

Supplemental Figure8. TNFα compensated for immune defects against *A. fumigatus* in neutrophils from IBT-treated patients. Human neutrophils from IBT-treated patients or healthy donors were incubated for 4h with TNFα at the indicated concentrations and co-incubated with *A. fumigatus* B5233 strain for all figure panels. **(A)** Neutrophils were incubated with *A. fumigatus* (MOI:0.25) for 5h, and a resazurin-based assay measured metabolic activity. Data calculated through time course study (see raw data in Supplemental Materials) and panel represents the output from linear regression analysis using Gompertz fit with percentages of growth inhibition of *A. fumigatus* by neutrophils in reference to the patient's untreated neutrophils. Error bars are 95% CI, $n = 3$, representative data from one IBT-treated patient. Ordinary one-way ANOVA and Tukey's multiple comparisons test with a single pooled variance demonstrated a p-value < 0.001 for TNFα treatments vs untreated, p-value = 0.0024 for 5 ng/mL TNFα, p-value = 0.0101 for 100 ng/mL TNFα. **(B)** Neutrophils were stimulated with 1 mg/mL *A. fumigatus* heat-killed hyphae. ROS production was measured by chemiluminescence using lucigenin. Error bars are SD, $n = 3$, representative data from one IBT-treated patient. **(C)** Neutrophils were co-incubated with labeled *A. fumigatus* swollen spores (MOI: 10). The percentage of phagocytic neutrophils ($CD45^+/CD66b^+/conidia^+$) was calculated based on the total number of viable neutrophils ($CD45^+/CD66b^+$) in each experiment. A minimum of 10,000 viable $CD66b^+$ events were recorded. Representative data from one IBT-treated patient.

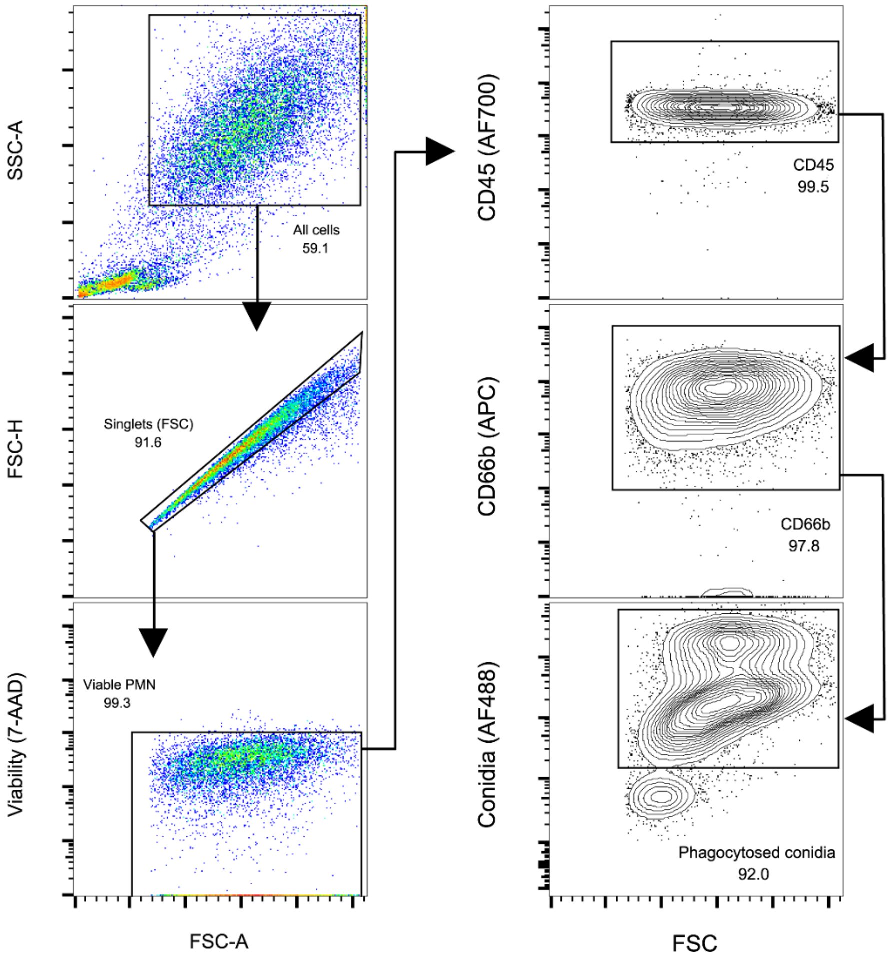

Supplemental Figure 9. Gating strategy for viable human neutrophils. Gating strategy for phagocytosis assay.
